# Quaking Inhibits Doxorubicin-Mediated Cardiotoxicity Through Regulation of Cardiac Circular RNA Expression

**DOI:** 10.1161/CIRCRESAHA.117.311335

**Published:** 2017-12-12

**Authors:** Shashi Kumar Gupta, Ankita Garg, Christian Bär, Shambhabi Chatterjee, Ariana Foinquinos, Hendrik Milting, Katrin Streckfuß-Bömeke, Jan Fiedler, Thomas Thum

**Affiliations:** From the Institute of Molecular and Translational Therapeutic Strategies (S.K.G., A.G., C.B., S.C., A.F., J.F., T.T.) and Excellence Cluster REBIRTH (T.T.), Hannover Medical School, Germany; Herz- und Diabeteszentrum NRW, Universitätsklinikum der Ruhr Universität Bochum, Erich und Hanna Klessmann-Institute for Cardiovascular Research and Development, Bad Oeynhausen, Germany (H.M.); Clinic for Cardiology and Pneumology, Stem Cell Laboratory, University Medical Center, Gottingen, Germany (K.S.-B.); DZHK (German Center for Cardiovascular Research) Partner site Göttingen, Germany (K.S.-B.); and National Heart and Lung Institute, Imperial College London, United Kingdom (T.T.).

**Keywords:** cardiotoxicity, doxorubicin, heart failure, noncoding RNA, RNA-binding protein

## Abstract

**Rationale:**

RBPs (RNA-binding proteins) have been described to be expressed and regulated in various organs including the heart. Little is known about the role of RBPs in heart failure induced by the chemotherapy drug doxorubicin and their interaction with circular RNAs.

**Objective:**

We aimed to identify key RBPs involved in doxorubicin-mediated heart failure and to elucidate their function.

**Methods and Results:**

Global transcriptome profiling from murine myocardium exposed to doxorubicin identified 5 differentially expressed RBPs. Expression of the RBP QKI (Quaking) in response to doxorubicin was strongly downregulated in rodent cardiomyocytes and human induced pluripotent stem cell–derived cardiomyocytes in vitro and in vivo in mice. Knockdown of *Qki* in primary cardiomyocytes increased apoptosis and atrophy after treatment with doxorubicin, whereas lentiviral mediated overexpression of *Qki5* inhibited the doxorubicin-induced apoptosis in cardiomyocytes. In vivo, AAV9 (adeno-associated virus serotype 9)–mediated cardiac overexpression of *Qki5* prevented cardiac apoptosis and cardiac atrophy induced by doxorubicin and improved cardiac function. Mechanistically, by lentiviral-based overexpression and CRISPR/Cas9-mediated silencing of *Qki5*, we identified regulated expression of specific circular RNAs derived from *Ttn* (Titin), *Fhod3* (Formin homology 2 domain containing 3), and *Strn3* (Striatin, calmodulin-binding protein 3). Moreover, inhibition of *Ttn*-derived circular RNA increased the susceptibility of cardiomyocytes to doxorubicin.

**Conclusions:**

We here show that overexpression of *Qki5* strongly attenuates the toxic effect of doxorubicin via regulating a set of circular RNAs. *Qki5* is, thus, an interesting target molecule to combat doxorubicin-induced cardiotoxicity.

## Abstract 2

Supplemental Digital Content is available in the text.

Cancer is a major public health concern worldwide, and its incidence is projected to rise because of an increasing age of the population. Chemotherapy drugs such as anthracyclines are often associated with cardiotoxicity leading to heart failure.^1^ In a retrospective analysis of 3 trials involving doxorubicin (an anthracycline class drug) treatment, 5%, 16%, and 26% of patients developed doxorubicin-mediated congestive heart failure at a cumulative dose of 400, 500, and 550 mg/m^2^, respectively.^2^ Despite its toxic effects, doxorubicin is frequently used in the clinics because of unavailability of any superior therapy. Therefore, there is an immediate need for the development of therapeutic strategies aiming at the reduction of doxorubicin-related cardiotoxicity without compromising its therapeutic function.

**Editorial, see p 188**

**In This Issue, see p 185**

**Meet the First Author, see p 186**

RNA, with the exception of tRNA and rRNA, not only was previously known to mainly serve as template for protein synthesis but also has regulatory function mainly through action of noncoding RNA species.^3^ Circular RNAs, a class of noncoding RNAs formed by back-splicing of exons, have recently been identified in eukaryotes with the help of deep sequencing and novel bioinformatics pipelines.^4^ Several reports demonstrate the presence of circular RNA molecules in various organs and cells including the heart.^5^ Several cardiac-specific genes like Titin and Ryanodine receptor 2 have been shown to form circular RNAs, a probable indication for a potential functional role.^5^ Recently, *Cdr1as*- and *Foxo3*-derived circular RNAs have been demonstrated to exert functional roles in the heart.^6,7^ Both coding or noncoding RNA (circular RNA) processing and function are dependent on RBPs (RNA-binding proteins).^3^

*Qki* (Quaking), an RBP of the signal transduction and activation of RNA family has been reported to regulate circular RNA formation during epithelial to mesenchymal transition.^8^ *Qki* is well known to regulate myelin formation in central and peripheral nervous system, but its cardiac function remains largely unknown.^9^ Quaking was found to inhibit ischemia/reperfusion-induced cardiomyocyte apoptosis by regulating *Foxo1* mRNA stability.^10^

Several RBPs are involved in cardiovascular pathophysiology, and their deletion in mice led to various cardiovascular abnormalities.^11^ Despite their involvement in various cardiac diseases, RBPs role in doxorubicin-associated cardiotoxicity remains unclear. Therefore, studies to understand the role of RBPs in doxorubicin-induced cardiotoxicity are needed.

Here, we performed transcriptome-wide analysis to identify RBPs involved in doxorubicin-induced cardiotoxicity. We identified an RNA-binding protein known as Quaking to be downregulated in response to doxorubicin. Furthermore, *Qki* deletion in cardiomyocytes increased their sensitivity to doxorubicin, whereas overexpression inhibited doxorubicin-induced apoptosis. Mechanistically, *Qki* inhibits doxorubicin-mediated cardiotoxicity via regulating cardiac circular RNAs.

## Methods

The authors declare that all supporting data are available within the article (and its Online Data Supplement).

### Animal Model

C57BL/6 N mice of 10 to 12 weeks of age were injected (intraperitoneally) with doxorubicin at a dose of 5 mg/kg once a week for consecutive 5 weeks. One week later after the last doxorubicin treatment, echocardiography data were recorded by an independent blinded researcher using the Vevo 2100 system (Fujifilm Visulasonics, Inc). Mice were euthanized, and hearts were harvested and processed for further molecular and cellular assays. Echocardiography data were analyzed using standard imaging protocols (M-mode and B-mode) for global cardiac volumes and functioning using the Vevostrain software (Fujifilm Visulasonics, Inc). Animal experiments were approved by the local authorities at Hannover Medical School and Niedersachsen Landesamt für Verbraucherschutz. AAV9 (adeno-associated virus serotype 9) was injected (intravenously) 1 week before first doxorubicin injection. Animal experiments were randomized and blinded with an internal number.

### Statistics

All data were analyzed using GraphPad Prism software. Data are presented as mean±SEM, and an unpaired 2-tailed *t* test was performed to calculate significance between 2 groups, and 1-way ANOVA with post hoc Tukey test was used to calculate significance difference between ≥3 groups wherever required.

Detailed method section can be found in the Online Data Supplement.

## Results

### Doxorubicin Downregulates Quaking Levels

**Figure 1.**
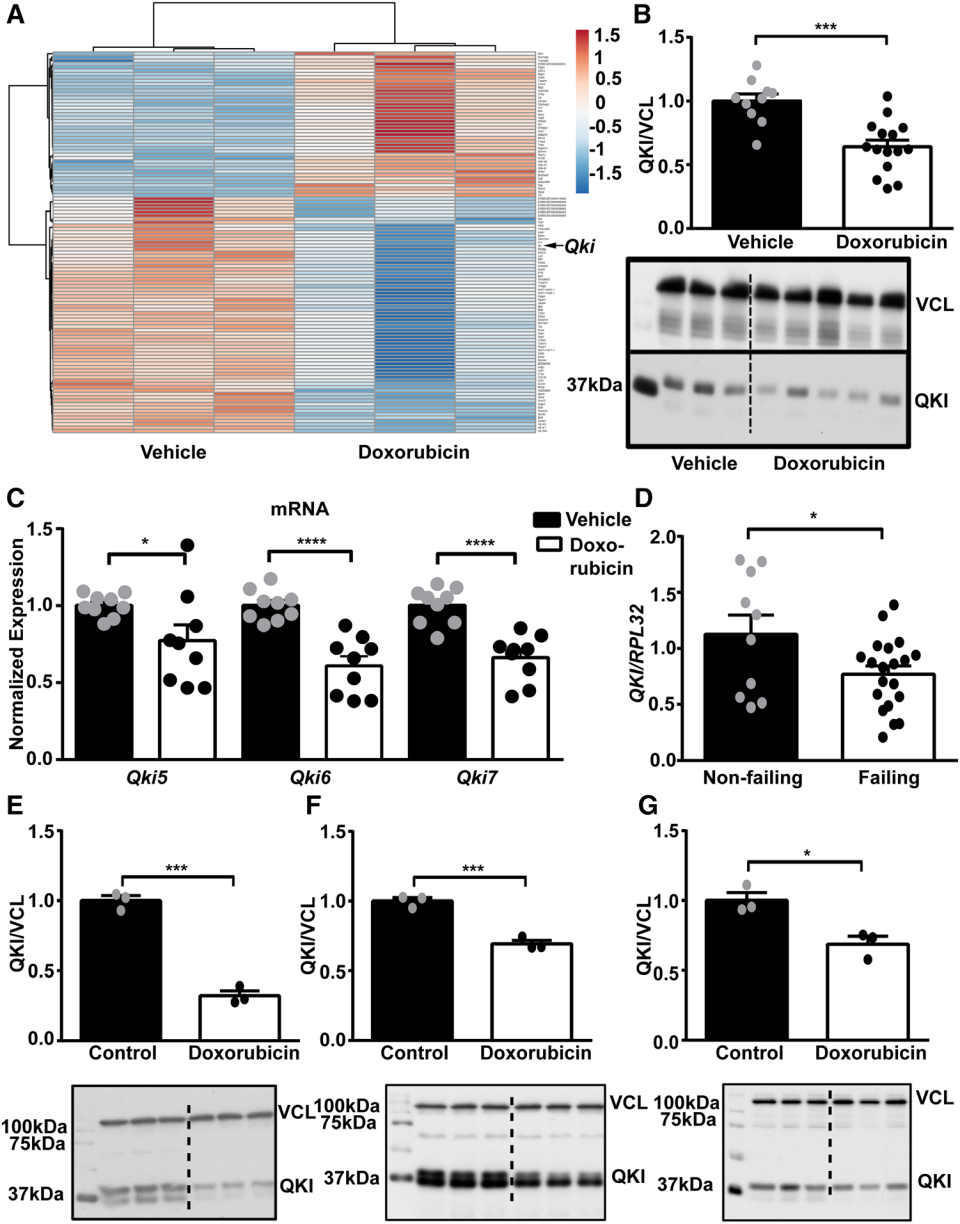
**Doxorubicin treatment reduced Qki (Quaking) expression**. **A**, Heatmap showing differentially expressed mRNAs in mouse myocardium treated with vehicle control or doxorubicin (n=3 each). *Qki* protein (**B**) and mRNA (**C**) levels in the myocardium of mice that had received doxorubicin compared with vehicle control ([**B**] n=10 vehicle and 15 doxorubicin and [**C**] n=9 vehicle and 9 doxorubicin). **D**, Expression of *QKI* in human failing heart compared with nonfailing heart (n=10 nonfailing and 20 failing). Doxorubicin (0.1 µmol/L) treatment for 72 h in HL-1 (**E**), neonatal rat cardiomyocytes (**F**), and human induced pluripotent stem cell–derived cardiomyocytes (**G**) significantly decreases Qki protein levels. *P≤0.05; **P≤0.01; ***P≤0.001; ****P≤0.0001.

We established an in vivo mouse model of doxorubicin-induced cardiotoxicity where we injected mice with doxorubicin (5 mg/kg) weekly for 5 consecutive weeks followed by scarification 1 week later (Online Figure IA). These mice showed clear signs of cardiac atrophy including reductions in heart weight to tibia length ratio, smaller cardiomyocyte cell sizes, and a significant decline in cardiac function as measured by echocardiography (Online Figure IB through ID; Online Table I). Electron microscopy confirmed myocardial damage as seen by less dense and destroyed myofibers in hearts of mice treated with doxorubicin (Online Figure IE). Global transcriptome profiling in cardiac tissue of doxorubicin-treated mice resulted in 113 differentially expressed mRNAs compared with vehicle control (Figure 1A; Online Table II). Because our aim was to identify RBPs involved in doxorubicin-induced cardiotoxicity, we compared the differentially expressed candidates with the complete list of mouse RBPs derived from the RBPDB (RNA-Binding Protein Database).^12^ Comparative analysis identified 5 RBPs among the dysregulated candidates. *Cirbp*, *Mkrn1*, *Rbm3*, and *Thumpd1* were found to be upregulated, whereas *Qki* was downregulated in response to doxorubicin. *Qki* was chosen for further studies because *Qki* was reported to inhibit cardiac apoptosis initiated by ischemia–reperfusion, and in addition, it is well known as a tumor suppressor gene.^9,10^ Therefore, we postulated that *Qki* overexpression would antagonize doxorubicin adverse effects on the myocardium and probably would not interfere with doxorubicin antitumor properties. First, we profiled protein levels of QKI in an organ panel of murine tissues and found abundant expression of QKI in brain followed by the heart (Online Figure IIA). Furthermore, we investigated expression of *Qki* mRNA in various cardiac cell types and found *Qki5* to be the most abundant isoform in the heart with highest expression in cardiomyocytes (Online Figure IIB). Similar to *Qki5*, *Qki6* was also enriched in cardiomyocyte fraction, whereas *Qki7* showed similar expression profiles in cardiomyocytes, fibroblasts, and endothelial cells (Online Figure II). Next, we validated our transcriptome approach and confirmed decreased protein and mRNA levels of all Quaking isoforms (*Qki5*, *Qki6*, and *Qki7*) in the myocardium after doxorubicin treatment (Figure 1B and 1C). Additionally, *QKI* mRNA was also found significantly downregulated in human failing compared with nonfailing hearts (Figure 1D). To study the significance of *Qki* on doxorubicin-induced cardiotoxicity and apoptosis, we treated HL-1, a mouse cardiomyocyte cell line, primary neonatal rat cardiomyocytes and human induced pluripotent stem cell–derived cardiomyocytes^13^ with doxorubicin. QKI levels were found to be significantly downregulated on treatment with doxorubicin in the HL-1 cardiomyocytes (Figure 1E), in primary rat cardiomyocytes (Figure 1F), and in human induced pluripotent stem cell–derived cardiomyocytes (Figure 1G). These results suggest an important role for quaking in cardiac pathophysiology.

### Quaking Exerts Protective Effects in Response to Doxorubicin

**Figure 2.**
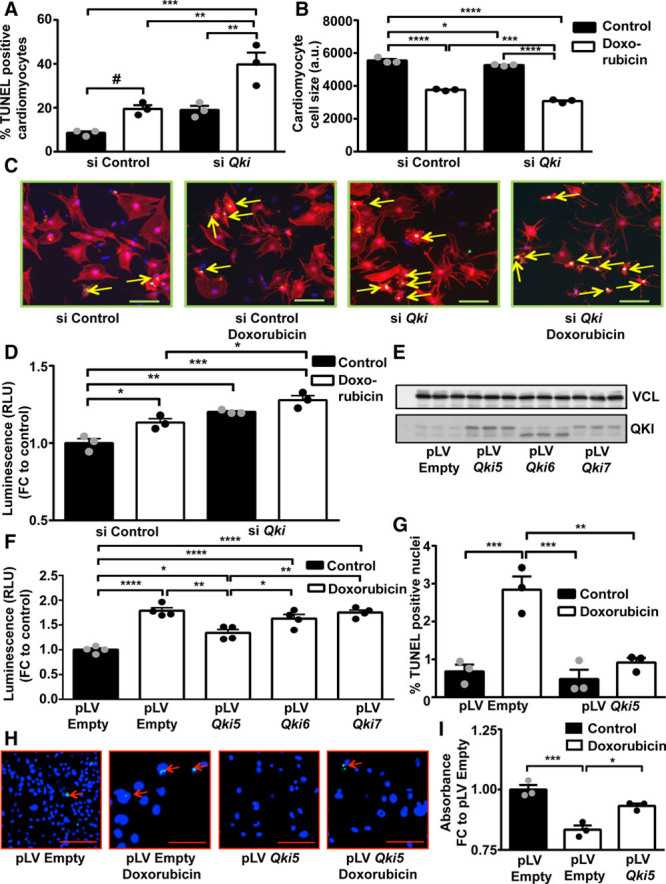
**Quaking exerts protective effects on cardiomyocyte exposed to doxorubicin**. Apoptosis measured by TUNEL (terminal deoxynucleotidyl transferase dUTP nick end labeling; green) staining (**A** and **C**) and cell size measurement (**B** and **C**) of positive primary cardiomyocytes (red—α-actinin sarcomeric) transfected with either siRNA control or siRNA *Qki5*, in presence or absence of doxorubicin. **D**, Caspase 3/7 activity in primary cardiomyocytes exposed to doxorubicin after inhibition of *Qki* or control. **E**, Western blot confirming lentiviral overexpression of *Qki* isoforms 5, 6, and 7 in HL-1 cells. **F**, Luminescence readings showing caspase 3/7 activity in different HL-1 pLV (plasmid Lentivirus) overexpression cell lines. **G–H**, Percentage of TUNEL (green)-positive nuclei in pLV empty and pLV *Qki5* overexpression cells with or without doxorubicin treatment. **I**, MTT (3-(4,5-dimethylthiazol-2-yl)-2,5-diphenyltetrazolium bromide) assay shows survival in HL-1 cells with Qki5 overexpression compared with controls after doxorubicin treatment. *P≤0.05; **P≤0.01; ***P≤0.001; ****P≤0.0001; #*P*=0.12. Bar=100 µm. a.u. indicates arbitrary unit; and RLU, relative luminescence unit.

To next test a potential functional role of *Qki* in cardiomyocytes, we used siRNAs to specifically knockdown *Qki* in primary rat cardiomyocytes (Online Figure IIIA). *Qki* silencing further induced apoptosis measured by an increased percentage of TUNEL (terminal deoxynucleotidyl transferase dUTP nick end labeling)-positive cardiomyocyte nuclei (Figure 2A and 2C) and led to increased cellular atrophy (Figure 2B and 2C) in the presence of doxorubicin. Furthermore, an increased caspase 3/7 activity was found in primary rat cardiomyocytes and H9C2 (rat myoblast cell line) cells after siRNA-mediated *Qki* inhibition compared with control in the presence of doxorubicin (Figure 2D; Online Figure IIIB). Because *Qki* was decreased on doxorubicin treatment and further inhibition of *Qki* increased susceptibility toward doxorubicin, we hypothesized that overexpression of *Qki* may inhibit cardiomyocyte apoptosis induced by doxorubicin. To test this, we generated HL-1 cell lines with specific overexpression of quaking isoforms (*Qki5*, *Qki6*, and *Qki7*) that are present in mice (Figure 2E) and subjected them to doxorubicin treatment. Doxorubicin treatment strongly increased apoptosis as measured by caspase 3/7 activity in control (pLV [plasmid Lentivirus] empty) cells, whereas apoptosis was significantly attenuated with *Qki5* overexpression, although little or no effects was seen with overexpression of *Qki6* or *Qki7*, respectively (Figure 2F). Additionally, we also performed TUNEL staining in HL-1 cells and found a decrease in the *Qki5*-overexpressing cell lines on treatment with doxorubicin compared with controls (Figure 2G and 2H). In line, an improved survival in *Qki5*-overexpressing cells in response to doxorubicin was observed (Figure 2I). Similarly, apoptosis staining by annexin V and 7-AAD (7-aminoactinomycin D) showed a trend for an inhibitory effect of *Qki5* on doxorubicin-induced apoptosis (Online Figure IIIC and IIID). Furthermore, AAV2 (adeno-associated virus serotype 2)-mediated *Qki5* overexpression in vitro (Online Figure IIIA) also attenuated doxorubicin-induced cardiac atrophy in neonatal rat cardiomyocytes (Online Figure IIIE and IIIF). These results indicated a protective effect of *Qki5* in doxorubicin-induced cardiotoxicity.

### Quaking Regulates Cardiac Circular RNAs

**Figure 3.**
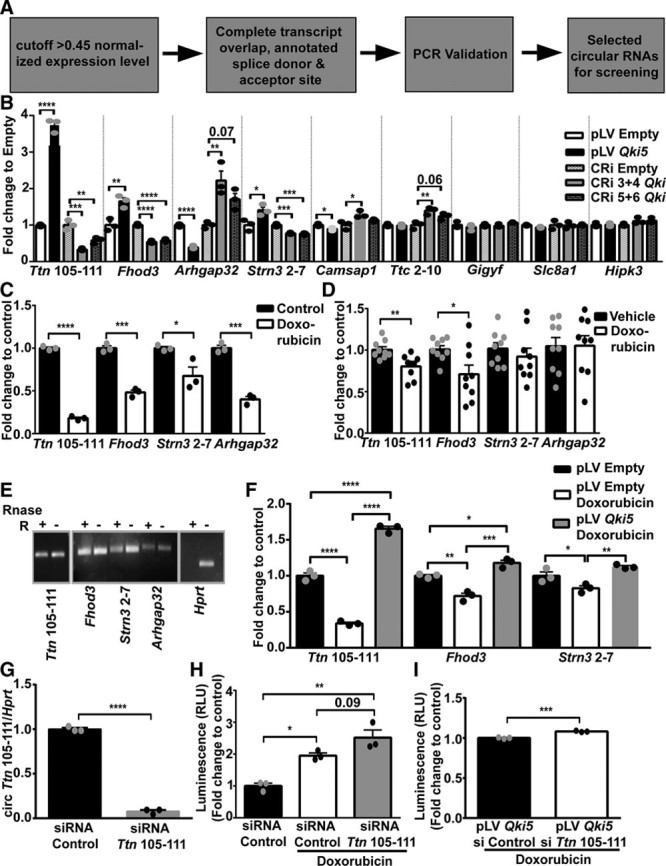
**Cardiac circular RNAs are regulated by Quaking**. **A**, Filtering strategy for screening of circular RNAs. **B**, Significantly changed expression levels of *Ttn* (Titin)-, *Fhod3* (Formin homology 2 domain containing 3)-, *Strn3* (Striatin, calmodulin-binding protein 3)-, and *Arhgap32*-derived circular RNAs in pLV (plasmid Lentivirus) empty, pLV *Qki5*, CRi empty, CRi 3+4 *Qki*, and CRi 5+6 *Qki* HL-1 cell lines. **C**, Circular RNAs derived from *Ttn*, *Fhod3*, *Strn3*, and *Arhgap32* expression in presence of doxorubicin. **D**, Expression of circular RNAs in murine myocardium exposed to doxorubicin (n=9 each). **E**, Circular RNAs and linear *HPRT* (hypoxanthine guanine phosphoribosyl transferase) polymerase chain reaction products from RNA treated with or without RNase R. **F**, Circular RNA expression levels in presence of doxorubicin are maintained with *Qki5* overexpression in HL-1 cells. **G**, siRNA-mediated knockdown of *Ttn* 104 to 110 circular RNA. **H**, Caspase 3/7 activity in response to doxorubicin on inhibition of *Ttn* 104 to 110 in HL-1 cells compared with control siRNA. **I**, Caspase 3/7 activity in pLV Qki5 cells after inhibition of circular RNA *Ttn* 104 to 111. *P≤0.05; **P≤0.01; ***P≤0.001; ****P≤0.0001. RLU indicates relative luminescence unit.

Previously, *Qki* was reported to regulate the formation of circular RNAs during epithelial to mesenchymal transition.^8^ Therefore, we decided to screen circular RNAs regulated by *Qki* in the heart to gain further mechanistic insights for the observed protective effect of *Qki5*. We selected a panel of highly expressed cardiac circular RNAs that we derived from previously published RNA-Seq data sets.^5^ An outline for our selection and filtering strategy is presented in Figure 3A. Details of the selected circular RNAs are presented in Online Table III. Selected circular RNAs were profiled in control (pLV empty) and pLV *Qki5*-overexpressing cell lines for their expression (Online Figure IVA). Additionally, we constructed a *Qki* knockdown HL-1 cell line with the CRISPR/Cas9 method (Online Figure IVB) and compared the expression of circular RNAs in 2 different knockdown cell lines (3+4 and 5+6 guide RNA combination) with the control (empty) cell line (Online Figure IVA). Candidate circular RNAs (*Ttn* [Titin] 105–111, *Fhod3* [Formin homology 2 domain containing 3], *Strn3* 2–7 [Striatin, calmodulin-binding protein 3], *Arhgap32*, *Camsap1*, *Ttc2* 2–10, *Gigyf*, *Slc8a1*, and *Hipk3*) (*Gene Name*, exon number), which showed a reciprocal regulation on overexpression of *Qki5* compared with CRISPR-mediated silencing of *Qki* were further investigated. We validated circular RNAs derived from the genes *Ttn*, *Fhod3*, and *Strn3* to be positively regulated with *Qki* expression, whereas *Arhgap32* was negatively regulated (Figure 3B). Thus, these results demonstrate that *Qki* regulates the expression of cardiac circular RNAs that may serve as potential downstream mediators for the observed protective *Qki5* effects. Next, we studied whether these circular RNAs would also be directly regulated by doxorubicin and indeed found that all of the circular RNAs were significantly downregulated with doxorubicin treatment in HL-1 cells (Figure 3C). Circular RNAs from *Ttn*, *Fhod3*, and *Strn3* were also downregulated in mouse hearts, which had doxorubicin-induced reductions in *Qki* levels, showing *Qki* as a likely upstream regulator (Figure 3D). Higher resistant to RNase R digestion before reverse transcription and polymerase chain reaction amplification confirmed that these candidates were indeed circular RNAs, whereas linear *Hprt* (hypoxanthine guanine phosphoribosyl transferase) was completely digested with RNase R treatment (Figure 3E). Furthermore, to confirm that these circular RNAs are downstream mediators of the *Qki5* protective effect, we evaluated their expression levels in pLV *Qki5*-overexpressing cells compared with control (pLV empty) cells in the presence of doxorubicin. Similar to previous results, *Ttn* 105–111, *Fhod3*, and *Strn3* 2–7 were downregulated in control (pLV empty) cells exposed to doxorubicin, whereas this reduction was totally absent in pLV *Qki5*-overexpressing cells (Figure 3F). We also checked the expression of these circular RNAs in *Qki6* and *Qki7* overexpression cell lines but only observed a modest increase confirming them to be specific targets of *Qki5* (Online Figure IVC). Next, we inhibited *Ttn* 105–111 circular RNA by a specific siRNA approach (Figure 3G) and evaluated its effect on doxorubicin-induced apoptosis. SiRNA-mediated inhibition of *Ttn* 105–111 led to increased susceptibility to doxorubicin as evident by higher caspase activity (Figure 3H). Additionally, the knockdown of *Ttn* 105–111 in pLV *Qki5*-overexpressing cell line also resulted in an induced caspase activity (Figure 3I). On the contrary, lentiviral mediated overexpression of circular RNA *Ttn* 105–111 resulted in lower caspase 3/7 activity and increased survival as seen by MTT (3-(4,5-dimethylthiazol-2-yl)-2,5-diphenyltetrazolium bromide) assay (Online Figure IVD though IVF). These results suggest that circular RNAs, especially derived from the titin gene, are downstream mediators of *Qki5*-mediated protective effects.

### Quaking Protects Hearts From Doxorubicin-Induced Cardiotoxic Effects

On the basis of our in vitro results, where we identified a beneficial effect of *Qki5* overexpression, we speculated that in vivo overexpression of *Qki5* could also reduce the doxorubicin-associated cardiotoxicity. To study the in vivo effects, we injected adult mice with 2*10^12^ AAV9 *Qki5* viral particles or controls. QKI5 expression was successfully increased in the hearts with AAV9 *Qki5* compared with controls (Online Figure VA). Contrary to our hypothesis, mice with AAV9 *Qki5* showed deteriorated cardiac function evident by declined ejection fraction, increased systolic and diastolic volume, and thinner interventricular septum thickness (Online Figure VB through VF). Moreover, TUNEL staining revealed increased apoptotic cells in the myocardium of the AAV9 *Qki5*-treated mice contradicting the in vitro findings (Online Figure VG and VH). However, RNA-binding proteins could be located in nucleus or cytoplasm to serve different purposes. *Qki5* has been shown to have nuclear localization signal and collectively transports other isoforms by heterodimerization.^14^ We hypothesized that probably alteration in localization of *Qki* could explain the contradicting results. Immunostaining of QKI in control mice hearts revealed that QKI is primarily located in the nucleus, whereas AAV9 *Qki5* hearts showed QKI localized both in the cytoplasm and nucleus (Online Figure VI). To quantify a dependency between the amount of QKI overexpression and resulting subcellular localization, we transduced neonatal rat cardiomyocytes with AAV2 *Qki5* at MOI (multiplicity of infection) of 5*10^3^ (low dose) and 1*10^5^ (high dose). QKI was localized mainly in the nucleus in the control and low-dose 5*10^3^ transduced cardiomyocytes, whereas the high dose of 1*10^5^ AAV resulted in mixed nuclear and cytoplasmic distribution as observed in the in vivo study (Online Figure VIA). Immunostaining of HL-1 cells revealed that QKI was primarily located in the nucleus in both pLV empty and *Qki5* overexpression cells (Online Figure VIB). Thus, we speculate that higher overexpression of *Qki5* results in additional cytoplasmic accumulation associated with more apoptosis. Therefore, along with overexpression of *Qki5*, its nuclear localization seems to be required to reverse the doxorubicin toxicity.

**Figure 4.**
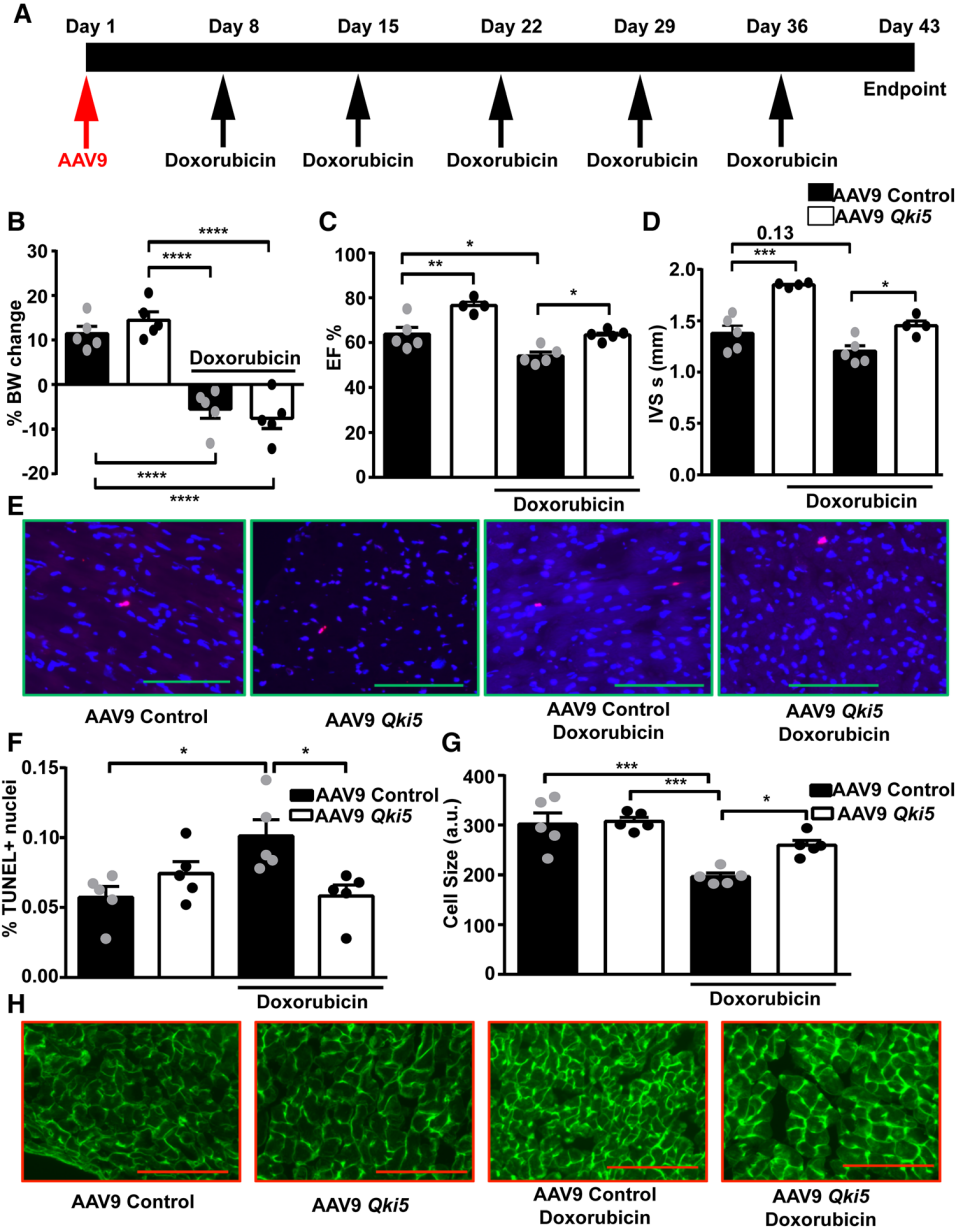
***Qki5* overexpression reverses doxorubicin-induced cardiotoxicity**. **A**, Schematic representation of the in vivo animal model and treatment scheme. **B**, Percentage body weight change during the experiment (n=5 each). Ejection fraction (**C**) and interventricular septum thickness (**D**) in AAV9 (adeno-associated virus serotype 9) control and AAV9 *Qki5*-treated mice with or without doxorubicin (n=4–5 each). Apoptosis measured by TUNEL (terminal deoxynucleotidyl transferase dUTP nick end labeling) staining in AAV9 control and AAV9 *Qki5* heart sections (**E** and **F**; n=5 each). Cardiomyocyte cell size measurement by Wheat germ agglutinin staining of heart (**G** and **H**; n=5 each). Bar=100 µm. *P≤0.05; **P≤0.01; ***P≤0.001; ****P≤0.0001. a.u. indicates arbitrary unit; BW, body weight; EF, ejection fraction; and IVS s, interventricular septum thickness systole.

On the basis of these results, we next tested moderate doses of AAV9 viral particles (7.5*10^11^) in the doxorubicin-induced cardiotoxicity model (Figure 4A). Here, QKI staining in hearts confirmed a preserved nuclear localization in AAV9 *Qki5*-treated mice (Online Figure VII). Doxorubicin led to similar decline in body weight in both the groups either with AAV9 control or AAV9 *Qki5* (Figure 4B). Contrary to high dose (2*10^12^) experiment, echocardiographic analyses of mice treated with low dose (7.5*10^11^) of AAV9 *Qki5* showed significantly improved ejection fraction and interventricular septum thickness, both under basal conditions and in response to doxorubicin (Figure 4C and 4D). Furthermore, percentage of TUNEL-positive cells was also significantly decreased in mice that received doxorubicin together with AAV9 *Qki5* compared with controls (Figure 4E and 4F). Doxorubicin-induced cardiac atrophy was also reversed in AAV9 *Qki5* group evident by significantly larger cardiomyocytes compared with control (Figure 4G and 4H). Of note, AAV9-based quaking dosing also increased cardiac ejection fraction under basal conditions, suggesting potential direct effects on contractility and hypertrophy; however, this novel aspect needs to be followed in future investigations. In conclusion, limited overexpression of *Qki5* reverses the doxorubicin-induced cardiotoxicity and confirms *Qki* to form a therapeutic target of interest.

## Discussion

RBPs that regulate the life cycle of RNA molecules are critical for cardiac biology.^11^ Here, we show that the RBP QKI, specifically the isoform *Qki5*, regulates cardiomyocyte apoptosis and atrophy induced by doxorubicin. The *Qki5* isoform has nuclear localization signal and is known to be responsible for nuclear translocation of other isoforms by heterodimer formation.^14^ Previously, Pilotte et al^14^ have reported that *Qki7* can function as apoptotic inducer when located in the cytoplasm. We found that strong overexpression of *Qki5* leads to both cytoplasmic and nuclear localization followed by massive apoptosis and cardiac dilatation. A possible explanation might be that too high expression of *Qki5* favors homodimerization and discourages heterodimerization, thus leaving *Qki6* and *Qki7* to stay in cytoplasm initiating apoptosis. In strong contrast, a modest overexpression of *Qki5* only resulted in nuclear localization and therapeutic effect in our doxorubicin-induced cardiotoxicity model. Thus, a narrow window exists for therapeutic utilization of *Qki5* via viral based therapeutic strategies. An alternative strategy would be to increase the expression of *Qki* from its endogenous loci through the CRISPR-dCas9 technology, which could simultaneously increase the expression of all isoforms without disturbing their stoichiometry and could lead to nuclear localization to exert potential beneficial effects. Another approach could be to identify downstream beneficial effectors of *Qki5* like circular RNAs and use them directly as therapeutic targets. Our findings confirm a central role of Quaking in the regulation of cardiac apoptosis and highlight its potential use as a therapeutic target with a narrow window, which could be improved via alternative strategies.

Deep sequencing and novel bioinformatics approaches led to the discovery of massive number of circular RNAs in the heart together with other organs.^5,15^ Despite their extensive presence, only few candidates like circular RNAs derived from *FoxO3* and *Cdr1* genes and *HRCR* were shown to regulate cardiac biology.^6,7,16^ Here, we report that circular RNAs derived from *Ttn*, *Fhod3*, and *Strn3* are regulated on doxorubicin treatment in the heart. Knockdown of a *Ttn*-derived circular RNA increased the susceptibility to doxorubicin, elucidating the functional role of circular RNAs in cardiotoxicity. Additionally, we identified Quaking as a key regulator of these cardiac circular RNAs. Quaking was previously shown to regulate circular RNA expression during epithelial to mesenchymal transition,^8^ but its role in the heart remained unexplored.

Indeed, we here discovered *Qki5* as a new target molecule to alleviate doxorubicin-induced cardiotoxic effects. Beneficial effects of a mild *Qki5* overexpression were mediated though cardiomyocyte apoptosis inhibition, although we cannot rule out other effects on hypertrophy or contractility at this stage. Mechanistically, our study demonstrates circular RNAs to serve as crucial mediators involved in the observed antiapoptotic effects. Further studies are warranted to understand the role of circular RNAs and their role as effector molecules in cardiac cells and as potential therapeutic candidates in other cardiovascular disease models.

## Acknowledgments

We acknowledge the technical help of Karina Zimmer and Dr Sabine Samolovac in animal experiments. We also acknowledge the help of Dr Sandor Batkai in writing the application for animal ethical permission for the in vivo experiment. We would also like to thank Dr Oliver Dittrich-Breiholz, Transcriptomics Facility, Hannover Medical School, for performing the mRNA arrays. We would also like to thank Dr Manoj Menon, Institute for Cell Biochemistry, Hannover Medical School, for his suggestions with cloning. We would also like to thank Dr Jan Hegermann and Gerhard Preiss, Institute of Functional and Applied Anatomy, Hannover Medical School, for their help with Transmission Electron Microscopy.

Author Contribution: S.K. Gupta developed the concept, designed the study, and wrote the initial manuscript draft. S.K. Gupta performed most of the experiments and analyzed the results. A. Garg, S. Chatterjee, and C. Bär provided help with AAV preparation. A. Garg also made the pLV *Ttn* 105–111 HL-1 cell lines. A. Foinquinos provided cardiac fractionated samples. J. Fiedler helped with the generation of CRISPR cell lines. K. Streckfuß-Bömeke provided the human induced pluripotent stem cell–derived cardiomyocytes. H. Milting performed the measurement of *Qki* in human heart samples. T. Thum provided his guidance during the study and helped with writing the manuscript.

## Sources of Funding

This study is funded by the Deutsche Forschungsgemeinschaft (DFG) GU 1664-1-1 grant to S.K. Gupta. T. Thum received funding from the IFB-Tx (BMBF 01EO1302), the REBIRTH Excellence Cluster, Fondation Leducq (MIRVAD [MicroRNA-Based Therapeutic Strategies in Vascular Disease]), and the European Union–funded ERC (European Research Council) Consolidator Grant LongHeart.

## Disclosures

T. Thum, J. Fiedler, and S.K. Gupta have filed and licensed patents about noncoding RNAs. T. Thum is founder of Cardior Pharmaceuticals GmbH. The other authors report no conflicts.

## Supplementary Material

## Novelty and Significance

### What Is Known?

- Doxorubicin, a commonly used chemotherapy drug, induces myocardial apoptosis and atrophy leading to cardiac malfunction, which limit its effective use in many cancer patients.
- RBPs (RNA-binding proteins) are known to play a crucial role in cardiac disease and development.
- Qukaing (*Qki5*) is an RBP that inhibits apoptosis induced by myocardial ischemia–reperfusion injury and regulates circular RNA formation during epithelial–mesenchymal transition.
