# Supplementary material for: Quaking Inhibits Doxorubicin-Mediated Cardiotoxicity Through Regulation of Cardiac Circular RNA Expression
Source: Circ Res. 2017 Dec 12;122(2):246–54. doi: 10.1161/CIRCRESAHA.117.311335 (PMC5771684; doi:10.1161/CIRCRESAHA.117.311335)
Supplement: Supplementary file 1 [file res-122-246-s001.pdf]

Supplemental Material

Quaking inhibits doxorubicin-mediated cardiotoxicity through regulation of cardiac circular RNA expression

Shashi Kumar Gupta PhD[1*], Ankita Garg M.S[1], Christian Bär PhD[1], Shambhabi Chatterjee M.Sc[1], Ariana Foinquinos PhD[1], Hendrik Milting PhD[2], Katrin Streckfuß-Bömeke PhD[3,4], Jan Fiedler PhD[1], Thomas Thum MD, PhD[1,5,6*]

Supplementary Methods

Cell culture & Reagents

HL-1 cells were cultured in Claycomb medium together with 10% FCS, norepinephrine, L-Glutamine and pencillin/streptomycin as per manufacturer's protocol. H9C2 cells were cultured in DMEM with 10% FBS and pencillin/streptomycin. Neonatal rat cardiomyocytes were isolated from one to three day old rat babies as previously described [1]. Human iPS derived cardiomyocytes were differentiated at the lab of Dr. Katrin Streckfuss-Bömeke as previously described [2]. All the cells were treated with 0.1µM Doxorubicin (Sigma-Aldrich) either for forty eight or seventy two hours. siRNA against Quaking (# rn.Ri.Qki.13.2 Integrated DNA Technologies) and custom designed circular RNA Ttn 105-111 (Qiagen) were transfected with Lipofectamine 2000 (Life Technologies) in OptiMEM medium.

Si Circular *Ttn* 105-111 – 5' GUUCAUGAAGGUGUCUAUUTT3'

Cardiac cell Fractionation

Cardiac cell fractionation was performed as described previously [3].

CRISPR Cas9-mediated knockdown of Quaking

Three guide RNAs were designed at exon 1 and exon 2 of the *Qki* mouse gene using http://crispr.mit.edu/ webpage and synthesized at EuroFins MWG. Guide RNAs were phosphorylated and annealed by incubation at 37°C for thirty minutes and then ramping 5°C every minute starting from 95°C until 25°C. Annealed oligos were cloned in LentiCRISPRv2 plasmid with BsmBI restriction digestion. Guide RNAs in LentiCRISPRv2, together with PAX2 and MD2.G plasmids were transfected in HEK293FT cells for lentivirus production. Cell supernatant was harvested and concentrated with Lenti-X Concentrator (ClonTech) and viral particles were collected next day. HL-1 cells were transduced with viral particles from two different guide RNAs in combination (1+2, 3+4 and 5+6). Three days later transduced cells were selected with puromycin. Knockdown of *Qki* was confirmed by western blot. The following sequences were used for guide RNAs

Exon 1-

Guide RNA 1 5'CACCGGGATCTTCAACCACCTCGAG 3'

Guide RNA 3 5'CACCGCAACCACCTCGAGCGGCTGC 3'

Guide RNA 5 5'CACCGCGTCCAGCAGCCGCTCGAGG 3'

Exon 2-

Guide RNA 2 5'CACCGACAATGGGTCCCACCGCGTC 3'

Guide RNA 4 5’CACCGGCAGAATTGCCTGACGCGGT 3’

Guide RNA 6 5’CACCGTGCAGAATTGCCTGACGCGG 3’

Caspase Assay

Caspase assay was performed with Caspase-Glo 3/7 kit (Promega) as per the manufacturer instructions. Briefly, cells were treated with 0.1µM Doxorubicn in normal medium with no FCS for forty eight hours. Next caspase assay reagent provided with kit was added in equal amount to the medium and incubated for thirty minutes at room temperature. Luminescence reading was measured at HT Synergy (Biotek) plate reader.

MTT Assaay

MTT assay was performed using Cell proliferation kit I (Roche) as per the manufacturer instruction. Briefly, HL-1 cells were seeded in 96 well plate and next day treated with 0.1µM Doxorubicin in normal medium without FCS for forty eight hours. Next, 10µL MTT reagent was added and incubated for four hours followed by addition of dissolving reagent and incubated overnight. Next day absorbance was measured at 580nm and 690nm at HT Synergy (Biotek) plate reader.

Annexin V & 7-AAD staining

Flow Cellect Annexin Red kit (Millipore) was used to stain apoptotic cells as per the manufacturer instruction. Briefly, cells were seeded in 24 well plate and next day treated with 0.1µM Doxorubicin in normal medium without FCS for forty eight hours. Then cells were trypsinized and harvested and stained with Annexin-V for 15 minutes at 37°C in 1X assay buffer. Annexin-V stained cells were centrifuged and washed with 1X assay buffer. Next cells were incubated with 7-AAD for 5 minutes and acquired on Guava (Millipore) flow cytometer. Data was analyzed with FLOWJO software.

TUNEL Staining

Cells were fixed with 4% paraformaldehyde for twenty minutes at room temperature and then permeabilized with ice-cold 0.1% Triton-X-100 in PBS for two minutes at room temperature. Next cells were incubated with enzyme labelling solution provided with In Situ cell death detection kit (Roche) for one hour at 37°C. For negative staining, enzyme was not added to the labelling solution. Cells were then washed and incubated with Dapi for 15 minutes. In case of neonatal rat cardiomyocytes, cells were stained with sarcomeric alpha-actinin (Sigma-Aldrich #A7811) after Tunel staining, in order to facilitate specific counting of Tunel positive cardiomyocytes. Images were taken with Nikon Eclipse Ti microscope and images were analyzed with NIS Elements. For analysis in each case, ten different images were analyzed from different regions and average value was taken. Three data points representing three individual experiments are shown.

Similarly, cryosections of heart were also processed for staining. Numerous images covering complete heart section was taken and Tunel positive nuclei were counted in each field. Average value for each heart was calculated.

Cell size measurement

Primary neonatal rat cardiomyocytes were seeded in 48 well plates, fixed with paraformaldehyde, permeabilized with 0.1% Triton-X-100 and stained with sarcomeric alpha-actinin (Sigma-Aldrich #A7811) and dapi. Images were taken with Nikon Eclipse Ti microscope and images were analyzed with NIS Elements. For analysis in each case, ten different images were analyzed from different regions and average value was taken. Three data points representing three individual experiments are shown.

Paraffin embedded or cryo preserved heart sections were stained with Alexa Fluor 488 labelled wheat germ agglutinin (Invitrogen) and dapi. Images were taken with Nikon Eclipse Ti microscope and images were analyzed with NIS Elements. For each heart six-ten different images were taken from different regions of the heart section and nearly two hundred to six hundred cells (depending upon the availability) area were measured and average value was taken for each heart.

Immunostaining

Neonatal rat cardiomyocytes or HL-1 cells were fixed with 4% paraformaldehyde for ten minutes and permeabilzed with 0.1% Triton-X-100 for 10 minutes. Blocking was done for thirty minutes with donkey serum and then incubated with QKI antibody (Sigma-Aldrich) (1:100) at 4°C overnight. Next day, cells were incubated with secondary antibody Anti-Rabbit-Alexa 594/488 (Invitrogen) and dapi for 30 minutes. Images were taken with Nikon Eclipse Ti microscope and images were analyzed with NIS Elements.

Similarly, cryosections of hearts were also stained for QKI.

Electron microscopy

All tissues were immersion-fixed in 150 mM HEPES, pH 7.35, containing 1.5% formaldehyde and 1.5% glutaraldehyde. Embedding and section preparation was done as before [4]at Institute of Functional and Applied Anatomy, Hannover Medical School. Images were at Transmission Electron Microscopy Morgagni 268 located in Central Electron Microscopy Facility, Hannover Medical School.

RNA Isolation & PCR

RNA isolation from cell culture and heart tissue was done using Trifast (Peqlab) as per the manufacturer’s instructions. Isolated RNA (500ng-1000ng) was reversed transcribed with random primer using iScript Select cDNA synthesis kit (Biorad). Real-Time quantative PCR was done with iQ SYBR Green mix (Biorad) on C1000 Touch Thermocycler (Biorad) using specific primer pairs listed in **Supplementary Table 4**. For amplification of circular RNAs, divergent primers were used while normal linear transcripts were amplified by convergent primers as usual. For RNase R resistant assay RNA was incubated with RNase R (Biozym Scientific) at 37°C for 10 minutes and heat activated at 95°C for three minutes. RNA was then reverse transcribed and amplified by specific PCR primers as mentioned before.

Circular RNAs screening were performed in pLV Empty, pLV *Qki5*, Cri Empty, Cri *Qki* 3+4 and Cri Qki 5+6 cell lines in triplicates. Validation was performed as n=3 individual experiments with three replicates each time.

Lentiviral overexpression cell lines

Qki5, *Qki6* and *Qki7* cDNA were synthesized as gene string from Thermo Fisher Scientific and cloned in pLV lentiviral backbone. Lentiviral overexpression of *Qki5*, *Qki6* and *Qki7* in HL-1 cells was mediated by transduction of lentivirus from pLV plasmid carrying cDNA sequence of *Qki5*, *Qki6*, *Qki7* and empty control. pLV plasmid were provided by Prof. Axel Sambach, Hannover Medical School. For lentiviral production, HEK293T were transfected with pLV plasmid together with helper plasmids (vsvg, gagpol, rev and NovB2) using CaCl2. Medium was changed after eight hours of transfection. Viral supernatant was collected forty eight to seventy two hours later. HL-1 cells were transduced with lentivirus and after three days puromycin selection was initiated to remove non-transduced cells. After puromyocin selection, overexpression was confirmed by western blot.

Similarly, exonic sequence of Ttn 105-111 circular RNA was synthesized as gene string from Thermo Fisher Scientific and cloned in circular RNA forming plasmid pCDNA3.1(+) ZKSCAN1 MCS exon vector (Addgene) with EcoRV and SacII. Then the insert together with repeat element responsible for circularization was released with digestion using KpnI and XhoI and cloned into pLV (lentiviral) plasmid. Virus production and permanent cell line were made similar to *Qki*.

Western Blotting

Cell pellet were lysed in 1X Cell lysis buffer (Cell Signaling) and isolated protein was measured by Bradford (Biorad) for quantification. 15 – 30 µg of protein was loaded for each sample on SDS-polyacrylamide gel to resolve the protein. Proteins were transferred from SDS PAGE gel to polyvinylidene fluoride membrane in Mini PROTEAN Tetra cell (Biorad). Specific proteins were identified by following antibodies: Quaking (Sigma #HPA019123) and Vinculin (Sigma #V9131). HRP conjugated secondary antibody (Cell Signalling) was used for detection of bands. Band intensity was calculated by Image J software.

Human Cardiac tissue samples

Human patient data was provided by Prof. Hendrik Milting. Heart tissue samples were obtained at Herz- und Diabeteszentrum NRW, Ruhr-Universität Bochum, Bad Oeynhausen. Patients gave informed consent for the use of their explanted hearts (ethical committee by the Medical Faculty of the Ruhr-University Bochum, suboffice Bad Oeynhausen). Failing group included ten patients each with ischemic cardiomyopathy and dilated cardiomyopathy, while non-failing included ten healthy donor hearts. Details of the patients are provided in **Supplementary Table 4**.

mRNA profiling

Microarray was performed on n=3 samples from each vehicle control and doxorubicin. The Microarray utilized represents a refined version of the Whole Mouse Genome Oligo Microarray 4x44K v2 (Design ID 026655, Agilent Technologies), called ‘048306On1M’ (Design ID 066423) developed at the Research Core Unit Transcriptomics (RCUT) of Hannover Medical School. Microarray design was created at Agilent’s eArray portal using a 1x1M design format for mRNA

expression as template. All non-control probes of design ID 026655 have been selected to be printed four times within a region comprising a total of 181560 Features (170 columns x 1068 rows). Four of such regions were placed within one 1M region giving rise to four microarray fields per slide to be hybridized individually (Customer Specified Feature Layout). Control probes required for proper Feature Extraction software operation were determined and placed automatically by eArray using recommended default settings.

250ng of total RNA were used for synthesis of aminoallyl-UTP-modified (aaUTP) cRNA with the 'Quick Amp Labeling kit, no dye' (#5190-0447, Agilent Technologies) according to the manufacturer's recommendations, except that reaction volumes were quartered and contained NTP-mix was exchanged by NTP Set (ATP, CTP, GTP, UTP) and aminoallyl-UTP (Fermentas, Thermo Scientific; order numbers R1091, R0481, respectively). Final NTP concentrations used for in-vitro transcription were 2.5mM (ATP, CTP, GTP) and 1.25mM (UTP, aaUTP). The labeling of aaUTP-cRNA was performed by use of Alexa Fluor 555 Reactive Dye (#A32756; LifeTechnologies) as described in the Amino Allyl MessageAmp™ II Kit Manual (#AM1753; Life Technologies) except that reaction volumes were quartered.

Prior to the reverse transcription reaction, 0.5µl of a 1:1000 dilution of Agilent's 'One-Color spike-in Kit stock solution' (#5188-5282, Agilent Technologies) were added to each 250ng of total RNA sample.

cRNA fragmentation, hybridization and washing steps were carried-out as recommended in the 'One-Color Microarray-Based Gene Expression Analysis Protocol V5.7', except that 800ng of each fluorescently labeled cRNA population were used for hybridization.

Slides were scanned on the Agilent Micro Array Scanner G2565CA (pixel resolution 3 µm, bit depth 20). Data extraction was performed with the 'Feature Extraction Software V10.7.3.1' using the extraction protocol file 'GE1_107_Sep09.xml', except that 'Multiplicative detrending' algorithm was inactivated.

Extracted signal intensities were filtered with cut-off of 2000 to remove low expressed mRNAs and global normalization was performed. Candidates with significant fold change of ≥ 1.5 were selected and heatmap was generated with Clustvis online tool.

Adeno-associated virus production

HEK 293T cells were transfected with AAV-Empty or AAV-Qki5 plasmid together with pDG (a kind gift from Prof. Roger Hajjar, Mount Siani Hospital, New York) (pDG2 for AAV2 and pDG9 for AAV9) with polyethylenimine. Medium was changed next day and cells were left for seventy two hours. Supernatant was collected and mixed with 40% Polyethylene Glycol 8000 solution for an hour and later incubated overnight at 4°C. Cells were harvested and lysed in presence of benzonase (Novagen). Supernatant was centrifuged at 2800xg for 15 minutes to collect precipitated virus and mixed with the cell lysate. Cell lysate was ultracentrifuged on iodixanol (OptiPrep, Progen) gradient in ultracentrifuge (Beckman Coulter) at 63000 rpm for one hour in Ti 70 rotor. AAVs were extracted from 40% fraction and concentrated using Amicon Ultra 15 (Millipore) columns. AAvs were titrated with PCR primer amplifying CMV (Cytomegalovirus) promoter.

References

1. Gupta SK, Foinquinos A, Thum S, Remke J, Zimmer K, Bauters C, de Groote P, Boon RA, de Windt LJ, Preissl S, Hein L, Batkai S, Pinet F, Thum T. Preclinical development of a MicroRNA-based therapy for elderly patients with myocardial infarction. *J Am Coll Cardiol*. 2016;68:1557-1571.

2. Streckfuss-Bomeke K, Wolf F, Azizian A, Stauske M, Tiburcy M, Wagner S, Hubscher D, Dressel R, Chen S, Jende J, Wulf G, Lorenz V, Schon MP, Maier LS, Zimmermann WH, Hasenfuss G, Guan K. Comparative study of human-induced pluripotent stem cells derived from bone marrow cells, hair keratinocytes, and skin fibroblasts. *Eur Heart J*. 2013;34:2618-2629.

3. Piccoli MT, Gupta SK, Viereck J, Foinquinos A, Samolovac S, Kramer FL, Garg A, Remke J, Zimmer K, Batkai S, Thum T. Inhibition of the cardiac fibroblast-enriched lncRNA Meg3 prevents cardiac fibrosis and diastolic dysfunction. *Circ Res*. 2017;121:575-583.

4. Rudat C, Grieskamp T, Rohr C, Airik R, Wrede C, Hegermann J, Herrmann BG, Schuster-Gossler K, Kispert A. Upk3b is dispensable for development and integrity of urothelium and mesothelium. *PLoS One*. 2014;9:e112112.

Online Figure I

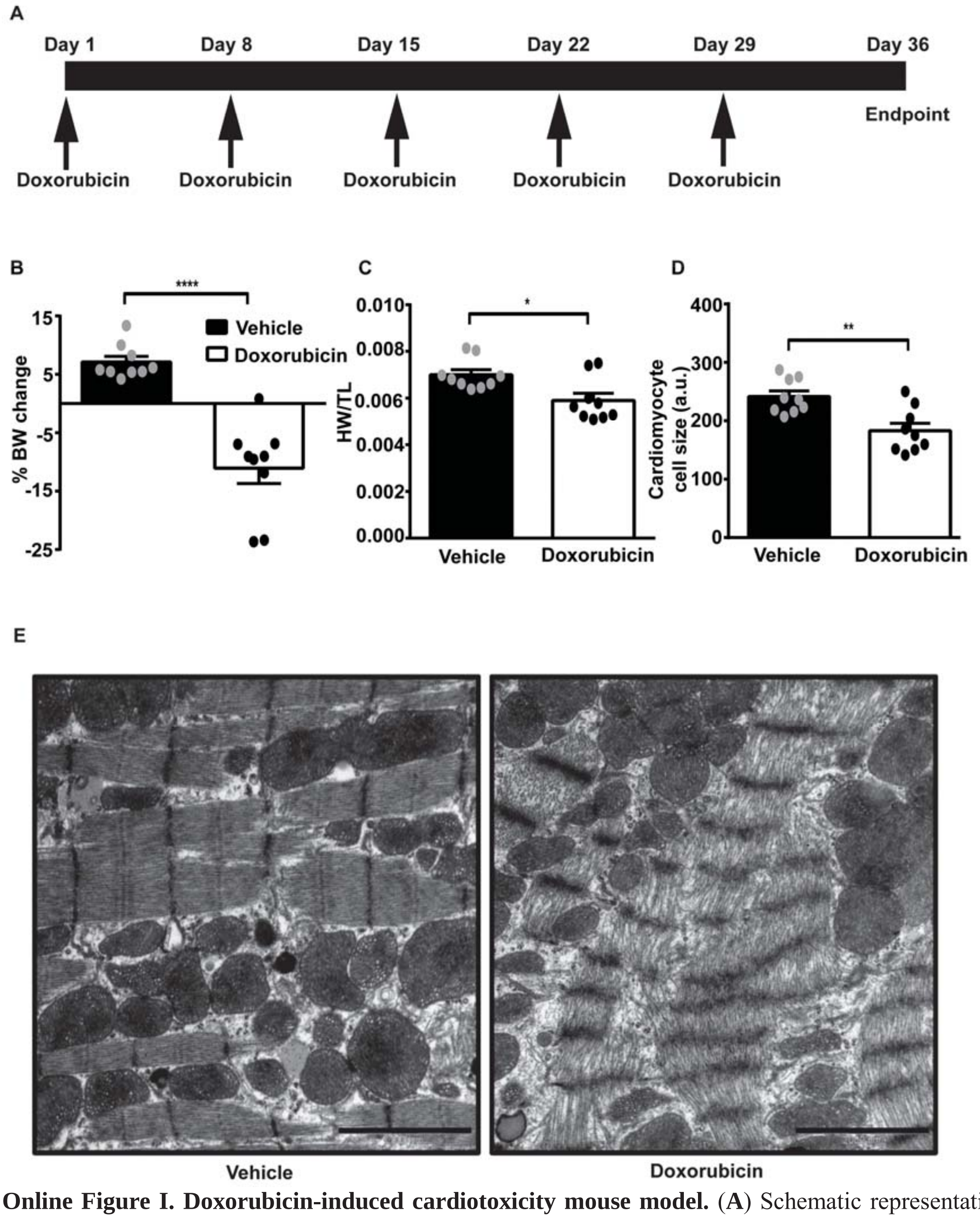

Online Figure I. Doxorubicin-induced cardiotoxicity mouse model. (**A**) Schematic representation of the *in vivo* animal model, where doxorubicin was injected weekly at 5mg/kg dose for five consecutive weeks. Body weight (**B**), heart weight to tibia length (**C**) and cardiomyocyte cell size (**D**) in animals which received doxorubicin compared to control (n=9 each). (**E**) Destroyed and less dense myofibres in doxorubicin treated mice were visualized by electron microscopy. *$p \leq 0.05$, **$p \leq 0.01$, ****$p \leq 0.0001$, a.u. arbitrary unit, Scale bar represents 2000nm

Online Figure II

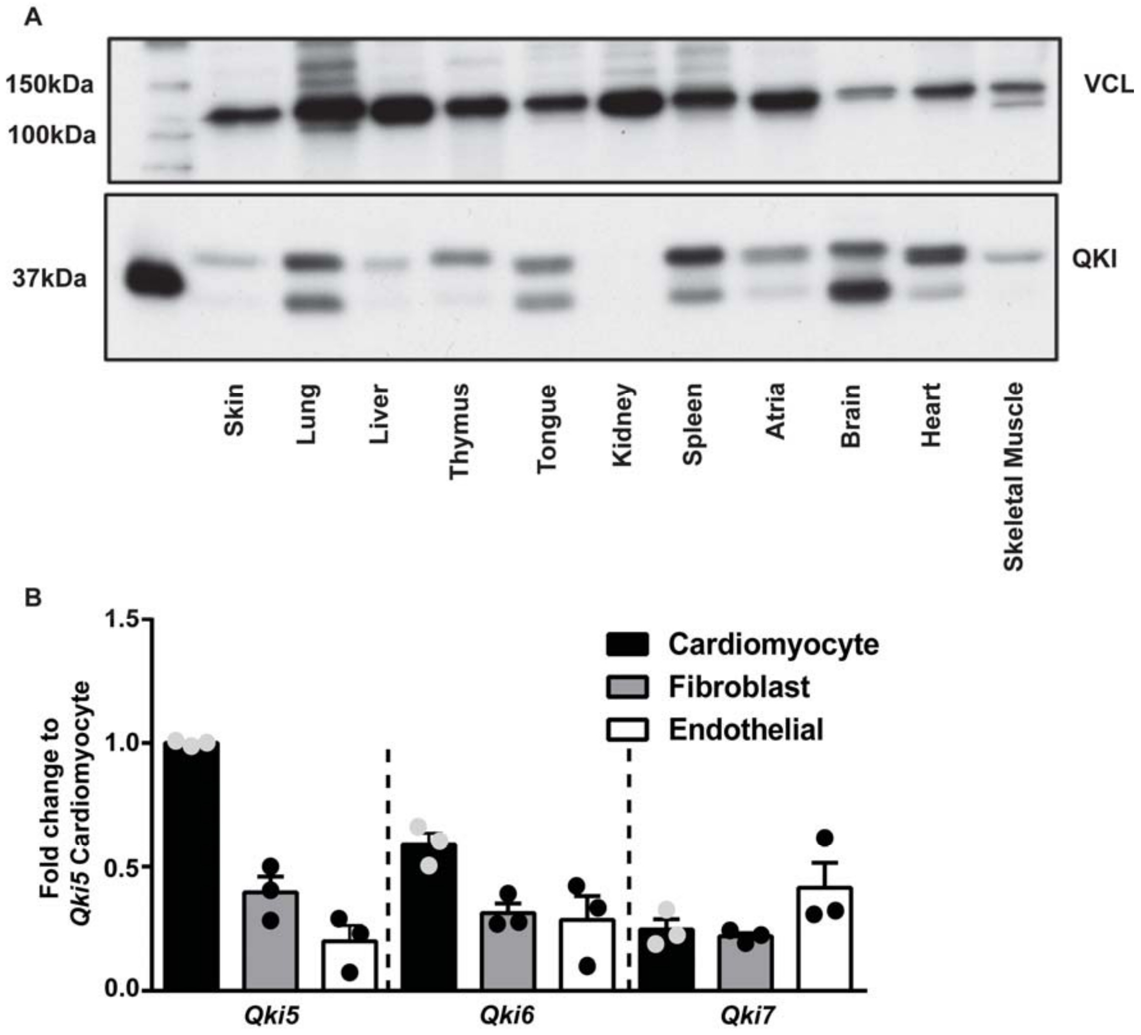

Online Figure II. (**A**) Expression of QKI in different mouse organs, (n=1). (**B**) Qki isoforms expression level in different cardiac cell types (n=3 each).

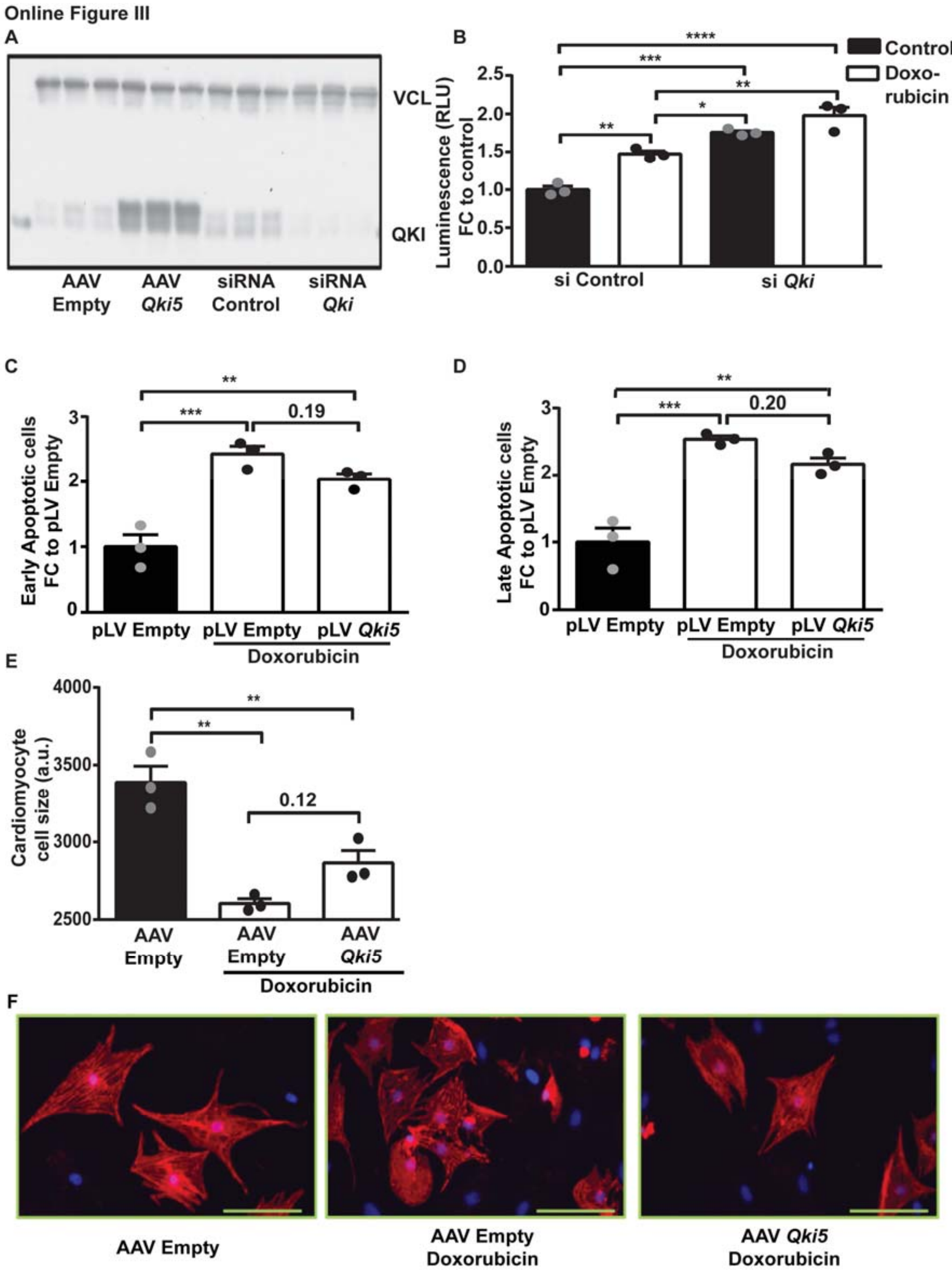

Online Figure III. Quaking modulation inhibits doxorubicin mediated cardiotoxicity. (**A**) AAV2 mediated overexpression of QKI5 and siRNA mediated inhibition of QKI in neonatal rat cardiomyocytes. (**B**) Caspase 3/7 activity in H9C2 cells transfected with siRNA against *Qki* or control in presence or absence of doxorubicin. (**C-D**) Annexin-V and 7-AAD staining of pLV Empty and pLV *Qki5* overexpressing cells, in response to doxorubicin. (**E-F**) Cell size measurements of cardiomyocytes transduced with AAV2 Qki5 or control vectors in the presence of doxorubicin. $^{**}p\leq0.01$, $^{***}p\leq0.001$, a.u. arbitrary units

Online Figure IV

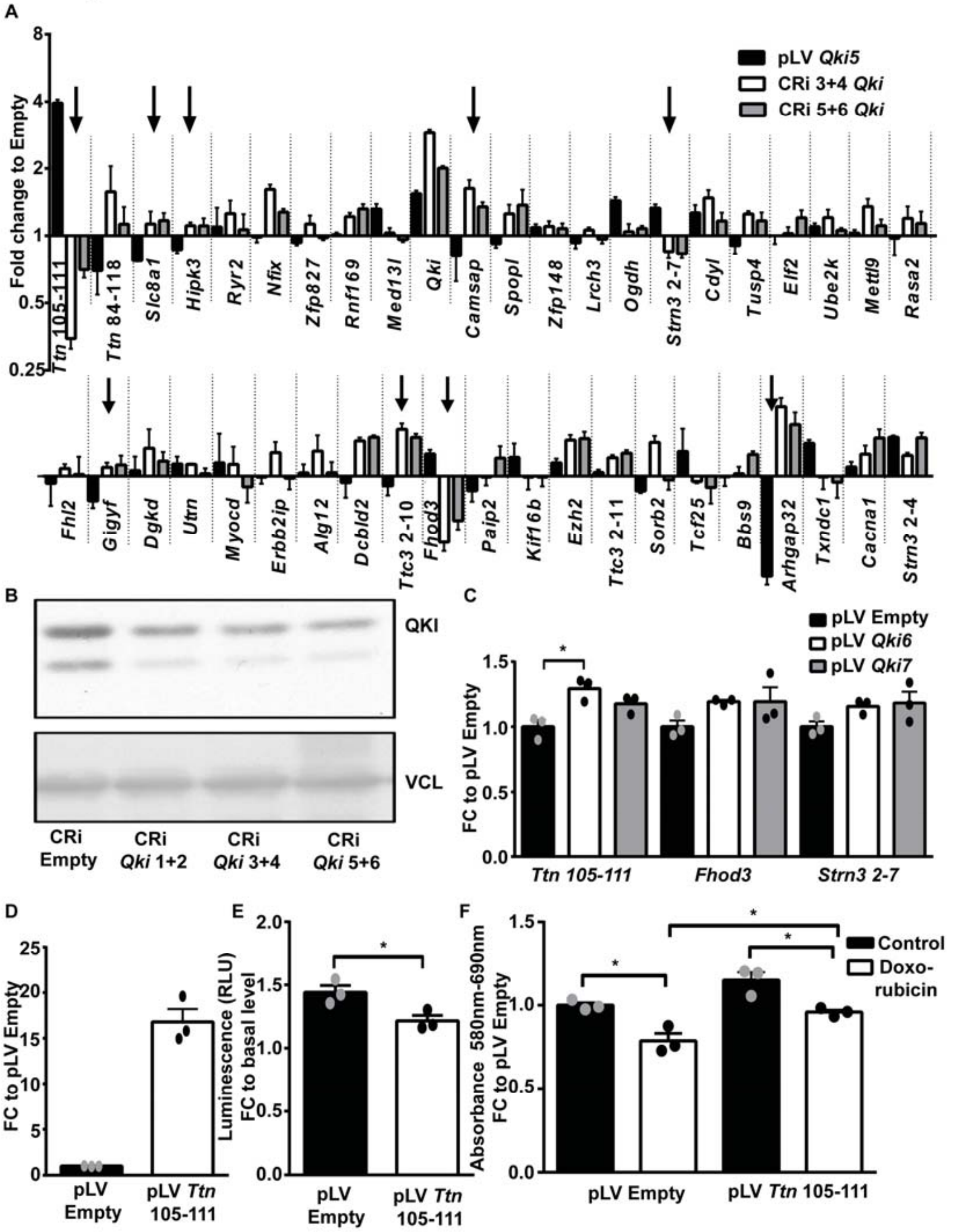

Online Figure IV. Screening of circular RNAs regulated by Quaking. Expression of circular RNAs selected after filtering in pLV Qki5, Cri 3+4 and Cri 5+6 HL-1 cell lines normalized to empty cell lines (**A**). Western blot showing CRISPR-mediated knockdown of Quaking (**B**). (**C**) Expression levels of circular RNAs derived from *Ttn*, *Fhod3* and *Strn3* in pLV *Qki6* and *Qki7* overexpressing cells. (**D**) Real-time PCR confirming overexpression of circular RNA *Ttn* 105-111 in HL-1 cells (**E**). Caspase3/7 activity in *Ttn* 105-111 overexpressing HL-1 cells compared to control upon doxorubicin treatment. (**F**) Cellular survival seen by MTT assay in *Ttn* 105-111 overexpressing HL-1 cells compared to pLV Empty controls. *p≤0.05, RLU – Relative Luminescence Unit, CRi - Crispr

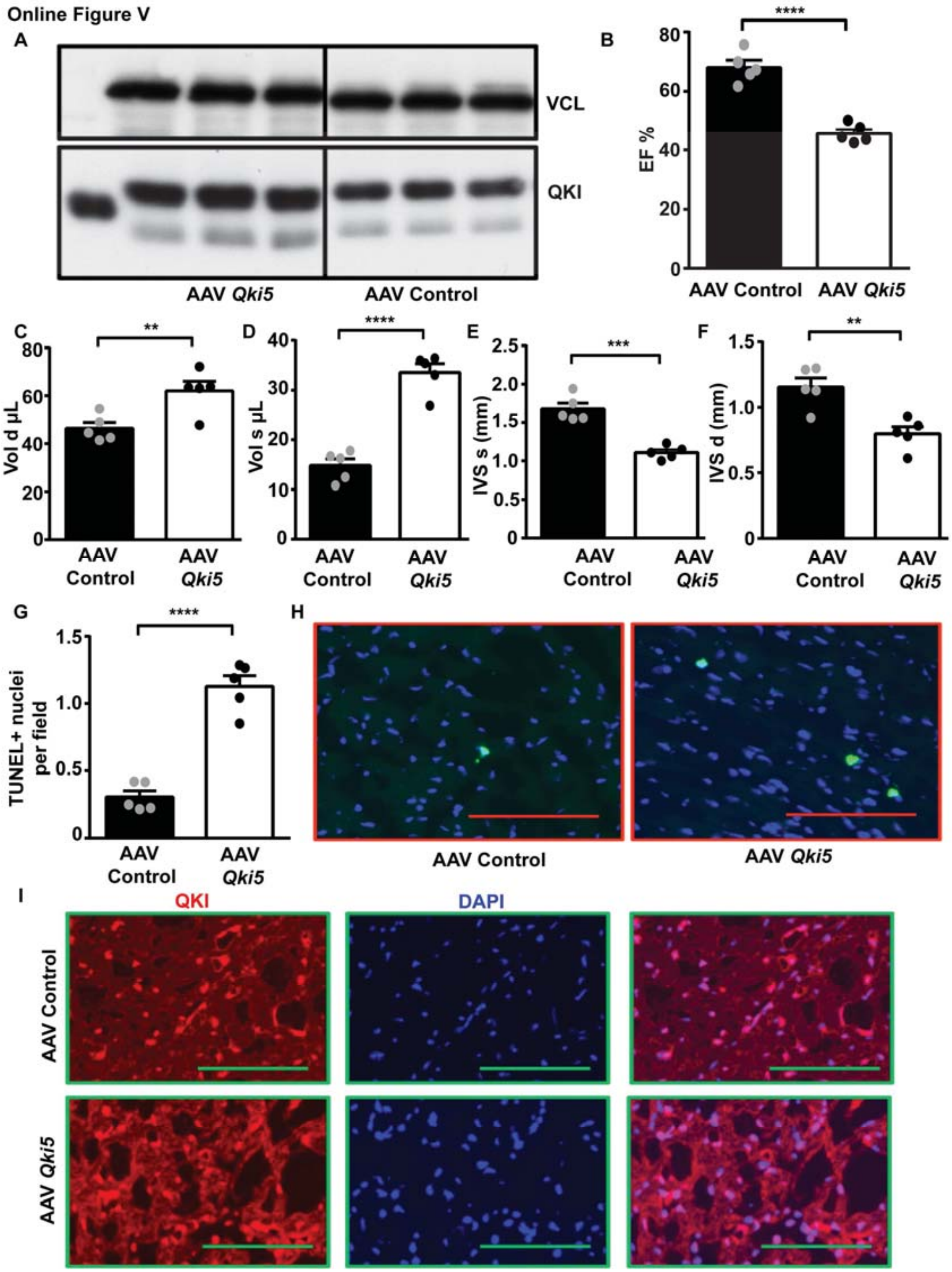

Online Figure V. Higher overexpression of *Qki5* leads to pronounced cytoplasmic localization and causes adverse effects in the heart. (**A**) Western blot experiments shows expression of QKI in myocardium of mice injected with AAV9-*Qki5* compared to control. (**B-F**) Cardiac function measured by echocardiography in AAV9-*Qki5* mice compared to control. (**G-H**) Higher number of apoptotic cells visualized by TUNEL staining in AAV9-*Qki5* treated myocardium. (**I**)Immunostaining of QKI in heart shows nuclear localization in control while AAV9-*Qki5* mice show distribution in cytoplasm and nucleus both. Scale Bar represents 100µM. $^*p \le 0.05$, $^{**}p \le 0.01$, $^{***}p \le 0.001$, $^{****}p \le 0.0001$, EF –

Ejection Fraction, Vol d – Volume diastole, Vol s – Volume systole, IVS s – Interventricular septum systole, IVS d – Interventricular Septum diastole

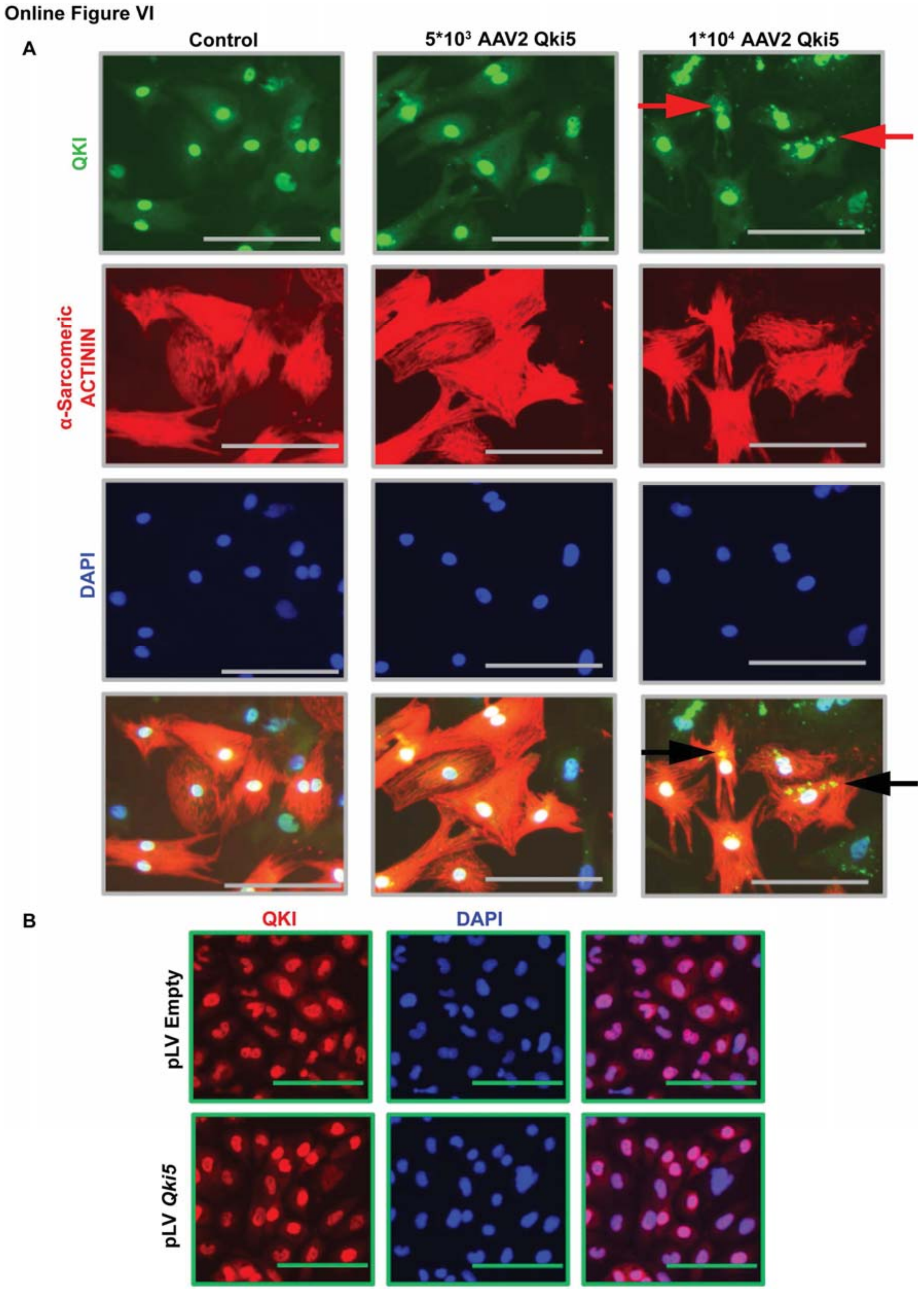

Online Figure VI. High amount of *Qki5* leds to cytoplasmic localization of QKI. (**A**) Transduction of neonatal rat cardiomyocytes with high dose (MOI-1*10^5) causes redistribution of QKI in cytoplasm and nucleus, while low dose (MOI-5*10^3) did not. (**B**) Quaking immunostaining in pLV empty and pLV *Qki5* overexpression cell lines shows nuclear localization of QKI. Scale Bar represents 100µM

Online Figure VII

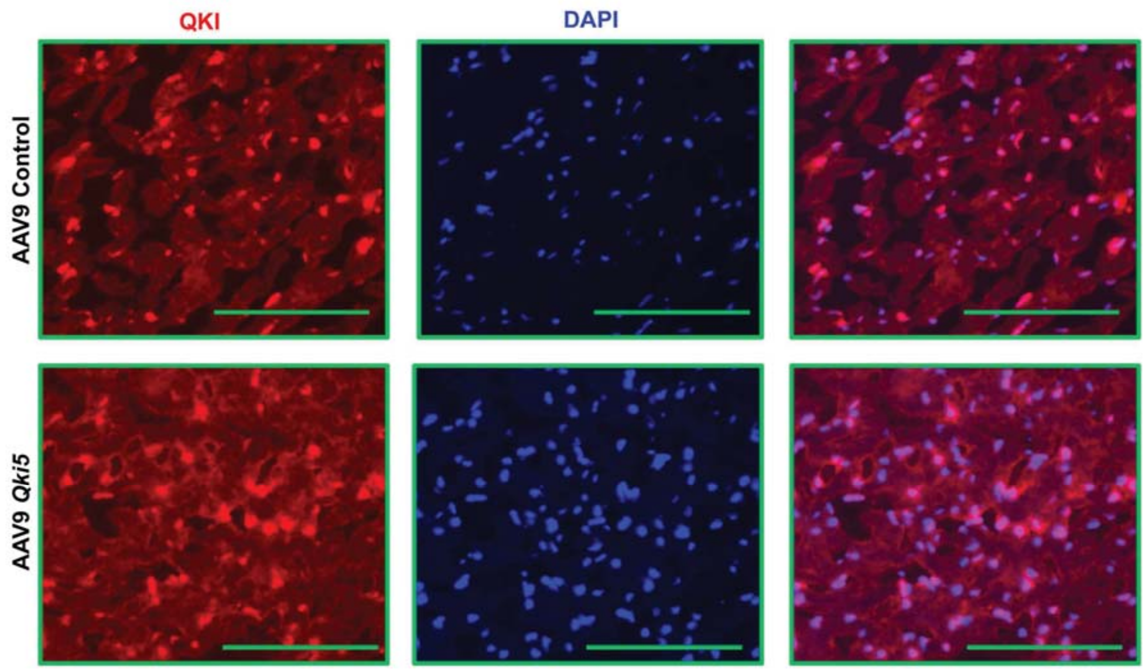

Online Figure VII. QKI staining in mice treated with 7.5*1011 AAV9 *Qki5* viral particles showed nuclear localization in heart similar to control hearts. Scale Bar represents 100µM.

	Vehicle n=5 (Mean±SEM)	Doxorubicin n=4 (Mean±SEM)	Significance
EF %	69.31 ± 1.534	47.98 ± 1.897	p< 0.0001
FS%	38.38 ± 1.170	23.84 ± 1.077	p< 0.0001
Vol s (µL)	19.61 ± 2.238	38.55 ± 4.255	p=0.0041
Vol d (µL)	63.25 ± 4.943	73.56 ± 5.491	p=0.2057
LVPW s (mm)	1.159 ± 0.0918	0.8602 ± 0.0673	p= 0.0414
LVPW d (mm)	0.8086±0.0762	0.6812 ± 0.0554	p=0.2400
IVS s (mm)	1.534 ± 0.0987	1.078 ± 0.0451	p=0.0064
IVS d (mm)	0.8391±0.0487	0.7277 ± 0.0211	p= 0.0975

Online Table I. Cardiac functions of Vehicle- and Doxorubicin- treated mice measured by echocardiography.

Downregulated Genes	Upregulated Genes
Acta1	Adamtsl4
Actg1	Alas2
Actg1	Amy1
Ankrd23	Art1
Ano10	Bpgm
Arl6ip5	Btg3
B2m	Camta1
Bcl6	Cdk5rap3
Bst2	Cirbp
C1qc	Cpt1a
Ccnd1	Ctgf
Cd93	Dbp
Col1a2	Eif4ebp1
Col3a1	ENSMUST00000040914
Col4a1	Gdap10
Cotl1	Hba-a1
Csf1r	Hbb-b2
Eef1e1	Hbb-bt
Emp1	Irf2bp2
ENSMUST00000099042	Itprip
ENSMUST00000099046	Ivns1abp
ENSMUST00000099050	Lrrc10
ENSMUST00000099683	Mcl1
ENSMUST00000099684	Mkrn1
ENSMUST00000173809	Mnt
ENSMUST00000177969	Nppa
Fabp5	Nr1d2
Fam131a	Nr4a1
Fn1	Pde1c
G0s2	Ptgds
Gm1987	Rbm3
H2-Eb1	Rit1
H2-K1	Scgb1c1
H2-K2	Serpinb6b
Hfe2	Slc25a37
HQ258995	Smim4
Hsph1	Tef
Ifi27	Thumpd1
Ift122	Timp4
Igsf1	Tns2
Igtp	Tspan4
Irgm2	Ttn
Lyz1	Usp2
Mal	
Marcks	
NAP111417-1	

NAP111439-1
NAP114472-1
Nrp2
Nrros
Nudt4
Pcsk6
Pgam1
Pi16
Plxnd1
Psmd14
Pxdn
Qk
Scn4b
Serpinh1
Sh3bgr
Sparc
Srpk2
Tmem242
Tmsb10
Tpm4
Traf7
Tuba1a
Ubiad1
Vim

Online Table II. List of mRNAs differentially regulated in doxorubicin treated hearts compared to vehicle control.

chr	start	end	strand	index	GeneSymbol	Annotation	nExons	length
chr1	43141698	43153254	-	mmuCirc_000119	Fhl2	CTO:ANNOTATED:CDS:5UTR	2	354
chr1	75497008	75514282	-	mmuCirc_000266	Obsl1	ITO:ALT_ACCEPTOR:CDS:5UTR	1	17275
chr1	87364104	87380008	+	mmuCirc_000298	Gigyf2	CTO:ANNOTATED:CDS	5	494
chr1	87880368	87881939	+	mmuCirc_000305	Dgkd	CTO:ANNOTATED:CDS	2	192
chr10	12436281	12455564	-	mmuCirc_000766	Utrn	CTO:ANNOTATED:CDS	5	725
chr11	6313788	6317080	+	mmuCirc_001237	Ogdh	CTO:ANNOTATED:CDS	3	386
chr11	59055377	59055640	-	mmuCirc_001406	Obscn	CTO:ANNOTATED:CDS	1	264
chr11	59055377	59061107	-	mmuCirc_001407	Obscn	CTO:ANNOTATED:CDS	3	792
chr11	59055961	59061107	-	mmuCirc_001409	Obscn	CTO:ANNOTATED:CDS	2	528
chr11	59055961	59061687	-	mmuCirc_001410	Obscn	CTO:ANNOTATED:CDS	3	792
chr11	65218529	65233184	-	mmuCirc_001466	Myocd	CTO:ANNOTATED:CDS	4	360
chr11	70874746	70884968	+	mmuCirc_001489	Rabep1	CTO:ANNOTATED:CDS:5UTR	2	333
chr12	51647994	51661713	-	mmuCirc_001998	Strn3	CTO:ANNOTATED:CDS	6	703
chr12	51655402	51661713	-	mmuCirc_002000	Strn3	CTO:ANNOTATED:CDS	3	260
chr13	11680966	11688013	-	mmuCirc_002359	Ryr2	CTO:ANNOTATED:CDS	4	427
chr13	35815909	35816575	+	mmuCirc_002451	Cdyl	CTO:ANNOTATED:CDS	1	667
chr13	103884143	103889285	-	mmuCirc_002718	Erbb2ip	CTO:ANNOTATED:CDS:5UTR	3	364
chr15	88811297	88812159	-	mmuCirc_003512	Alg12	CTO:ANNOTATED:CDS	2	328
chr16	11116795	11128703	-	mmuCirc_003625	Txndc11	CTO:ANNOTATED:CDS	3	445
chr16	32950292	32961744	+	mmuCirc_003757	Lrch3	CTO:ANNOTATED:CDS	5	625
chr16	33421321	33434974	+	mmuCirc_003769	Zfp148	CTO:ANNOTATED:CDS:5UTR	3	567
chr16	58424561	58433463	+	mmuCirc_003842	Dcbld2	CTO:ANNOTATED:CDS	2	366
chr16	94383912	94403368	+	mmuCirc_003895	Ttc3	CTO:ANNOTATED:CDS:5UTR	10	911
chr17	6137211	6139156	+	mmuCirc_003928	Tulp4	CTO:ANNOTATED:CDS:5UTR	1	1946
chr17	10238893	10282981	-	mmuCirc_003940	Qk	CTO:ANNOTATED:CDS	3	404
chr17	34715683	34797443	+	mmuCirc_004045	Tnxb	ITO:ALT_DONOR:CDS:3UTR	1	81761
chr17	34716301	34798563	+	mmuCirc_004046	Tnxb	ITO:ALT_DONOR:CDS:3UTR	1	82263
chr17	81647809	81649638	-	mmuCirc_004295	Slc8a1	CTO:ANNOTATED:CDS:5UTR	1	1830
chr18	5633204	5705243	+	mmuCirc_004341	Zeb1	CTO:ALT_ACCEPTOR:CDS	1	72040
chr18	25020662	25028169	+	mmuCirc_004438	Fhod3	CTO:ANNOTATED:CDS	3	558
chr18	35610871	35613502	+	mmuCirc_004508	Paip2	CTO:ANNOTATED:CDS:5UTR	2	344
chr2	23537328	23545579	-	mmuCirc_005197	Spopl	CTO:ANNOTATED:CDS:5UTR	7	869
chr2	25965663	25966948	-	mmuCirc_005227	Camsap1	CTO:ANNOTATED:CDS	2	425
chr2	70770004	70786995	-	mmuCirc_005427	Tlk1	CTO:ANNOTATED:CDS	2	191
chr2	76857904	76881839	-	mmuCirc_005504	Ttn	CTO:ANNOTATED:CDS	34	6213
chr2	76857904	76884197	-	mmuCirc_005505	Ttn	CTO:ANNOTATED:CDS	35	6501
chr2	76863274	76868085	-	mmuCirc_005516	Ttn	CTO:ANNOTATED:CDS	7	780
chr2	104470749	104471847	-	mmuCirc_005592	Hipk3	CTO:ANNOTATED:CDS:5UTR	1	1099
chr2	131511984	131516443	+	mmuCirc_005746	Smox	CTO:ANNOTATED:CDS:5UTR	2	461
chr2	142739523	142740991	-	mmuCirc_005775	Kif16b	CTO:ANNOTATED:CDS	2	172
chr3	51308050	51326035	-	mmuCirc_006048	Elf2	CTO:ANNOTATED:CDS:5UTR	3	484
chr5	65565989	65594548	+	mmuCirc_007206	Ube2k	CTO:ANNOTATED:CDS	5	465
chr5	118593333	118593570	+	mmuCirc_007425	Med13l	CTO:ANNOTATED:CDS	1	238
chr6	47576536	47577667	-	mmuCirc_007816	Ezh2	CTO:ANNOTATED:CDS:5UTR	2	253

chr6	119052601	119057515	-	mmuCirc_008045	Cacna1c	CTO:ANNOTATED:CDS	2	428
chr7	99935162	99955540	-	mmuCirc_008473	Rnf169	CTO:ANNOTATED:CDS	3	340
chr7	121047838	121057399	+	mmuCirc_008575	Mettl9	CTO:ANNOTATED:CDS	3	586
chr8	11212664	11241198	-	mmuCirc_008719	Col4a1	CTO:ALT_DONOR:ALT_ACCEPTOR:CDS	31	3111
chr8	45759053	45775703	+	mmuCirc_008813	Sorbs2	CTO:ANNOTATED:CDS	6	714
chr8	79118175	79136663	+	mmuCirc_008936	Zfp827	CTO:ANNOTATED:CDS	3	402
chr8	84771784	84772315	-	mmuCirc_008966	Nfix	CTO:ANNOTATED:CDS	1	532
chr8	95365314	95366422	+	mmuCirc_009025	Mmp15	CTO:ANNOTATED:CDS	2	278
chr8	123382527	123383443	+	mmuCirc_009109	Tcf25	CTO:ANNOTATED:CDS	2	194
chr9	14571623	14575439	-	mmuCirc_009167	Amotl1	CTO:ANNOTATED:CDS	3	524
chr9	22887581	22887996	+	mmuCirc_009200	Bbs9	CTO:ALT_DONOR:CDS:3UTR	2	243
chr9	32246447	32250787	+	mmuCirc_009220	Arhgap32	CTO:ANNOTATED:CDS	5	679
chr9	96591950	96611499	-	mmuCirc_009595	Rasa2	CTO:ANNOTATED:CDS	4	394
chr9	96602713	96611499	-	mmuCirc_009596	Rasa2	CTO:ANNOTATED:CDS	3	317

Online Table III. Detailed list of circular RNAs selected for screening after our filtering criteria mentioned in the **Figure 3A**.

Gene name		
Circ_Ttn 104-110	Fwd primer 5’ AAGAGGGCTACGATGAAGGG 3’	Rev primer 5’ GTACAGTTCCGCTGTGCTTC 3’
Circ_Ttn 84-118	Fwd primer 5’ CAAAGAAACCTGCTCCCGAAG 3’	Rev primer 5’ ATCGGTGCTGTTCCAGTGACAT 3’
Circ_Slc8a1	Fwd primer 5’ TCTGGAGCTCGAGGAAATGT 3’	Rev primer 5’ TTGGGTGGGAGACTTAATCG 3’
Circ_Hipk3	Fwd primer 5’ CATGCTGACCTCAAACCAGA 3’	Rev primer 5’ ACACAACCGCTTGGCTCTAC 3’
Circ_Ryr2	Fwd primer 5’ CCTCTCTGCACTGGAGGACAT 3’	Rev primer 5’ CCTTGGCTGTCAGTGTGTCAT 3’
Circ_Nfix	Fwd primer 5’ CTCGAACCCCGGCCTGTGT 3’	Rev primer 5’ GGTTGAACCAGGTGTAGGAGA 3’
Circ_Zfp827	Fwd primer 5’ CACCTCCAATTCAAAAGATCTGC 3’	Rev primer 5’ ATGTTCCGGCTGGGTGATAT 3’
Circ_Rnf169	Fwd primer 5’ CCAGATGATGCAGACCCATC 3’	Rev primer 5’ CTTTCTCAGGCATCCATACTCC 3’
Circ_Med13l	Fwd primer 5’ GGATATTCTGGTGGGGAGATGA 3’	Rev primer 5’ GAAACTTAACAGGATTGGATCATCTT 3’
Circ_Qki	Fwd primer 5’ GCCCAATTGGGAGCATCTAA 3’	Rev primer 5’ CACCGCGTCAGGCAATTCT 3’
Circ_Camsap	Fwd primer 5’ GGCCAGTGTCAAGCGCTTTT 3’	Rev primer 5’ AGAAGCTTGATAACGGGTGG 3’
Circ_Spopl	Fwd primer 5’ CTCGATCCCCAGTTTTTAATGC 3’	Rev primer 5’ AGGCAGAGGTGGAGTAGGTT 3’
Circ_Zfp148	Fwd primer 5’ GAGCAGATGGACACCCACGA 3’	Rev primer 5’ TGTGTCAGGTATAAGCCCATCC 3’
Circ_Lrch3	Fwd primer 5’ TACCTGAACATACAAGCTTGTAAG 3’	Rev primer 5’ TGGGATATACCGAATACAATTCTGG 3’
Circ_Ogdh	Fwd primer 5’ GGATGCTGATCTGGACTCCT 3’	Rev primer 5’ GCATGGGCCATGGTAGCCAG 3’
Circ_Strn3 2-7	Fwd primer 5’ GATCCAGATACTGAGGAAGCAC 3’	Rev primer 5’ TCTTCAAATTCTCTTGACCTTTCC 3’
Circ_Cdyl	Fwd primer 5’ AGGTTTCTGGCCCCGTGACT 3’	Rev primer 5’ CCAGATATTCTGTCTTCCCTTTCTTG 3’
Circ_Tusp4	Fwd primer 5’ TCGAGTCACTGTCGCAGAGA 3’	Rev primer 5’ TAGACAGAGTCTTCAAATGGCAC 3’
Circ_Elf2	Fwd primer 5’ TTAGAGCAGGGCTATGCTGC 3’	Rev primer 5’ TAGAGATGGAGTAGACATCCGG 3’
Circ_Ube2k	Fwd primer 5’ GTTTCTAGTCCAGAATACACCAA 3’	Rev primer 5’ GGTGTGTCTGGAGGTCCTGC 3’
Circ_Mettl9	Fwd primer 5’ GCAGGGTCATCCTGGCATT 3’	Rev primer 5’ GAAGATCTGTGTTCCTTGGTC 3’
Circ_Rasa2	Fwd primer 5’ GAGTTAAGACTGAATGAGCTGA 3’	Rev primer 5’ GCGATAAACTTCTTCCTGGTCC 3’
Circ_Fhl2	Fwd primer 5’ GGTGGACAAGCCCTTTGCTG 3’	Rev primer 5’ ATGTACTTCTTGCCGTACAGAG 3’
Circ_Gigyf	Fwd primer 5’ AGGGGTGACAGGCGCTTTG 3’	Rev primer 5’ CTCGGCCATATCGATAATCTGCT 3’
Circ_Dgkd	Fwd primer 5’ GATGAGGTAGACCTGACTGATG 3’	Rev primer 5’ CATAGTAAAGTGTTCGCCCTCG 3’
Circ_Utrn	Fwd primer 5’ ATGATGTCTGCCAGAGTTGC 3’	Rev primer 5’ GGACACTCAGGAGCTGATCAT 3’
Circ_Myocd	Fwd primer 5’ GAAGATCGCTCTCCGCCCAG 3’	Rev primer 5’ CTTGGTTAGCCAGCTGCTCC 3’
Circ_Erbb2ip	Fwd primer 5’ CATTACCAGCCTCCATTGCA 3’	Rev primer 5’ CGACATGGTACCAACCGCAC 3’
Circ_Alg12	Fwd primer 5’ CTGCGGTTCATCATCTATACCT 3’	Rev primer 5’ CCACTCCTTCCGGCCATACA 3’
Circ_Dcbld2	Fwd primer 5’ TCACAGTGCTGTTCATGAGTG 3’	Rev primer 5’ AGTTGATGGATGTAAGGGTTCC 3’
Circ_Ttc3 2-10	Fwd primer 5’ GGCAGTTTAGGACTGTGCAG 3’	Rev primer 5’ TCCTCATAAGGGTAATCTTCCAG 3’
Circ_Fhod3	Fwd primer 5’ CAGCAGCAACTCTTTCCAATCC 3’	Rev primer 5’ CCTCCTCTGAATTGGGCTCC 3’
Circ_Paip2	Fwd primer 5’ CGAGATCTCCCACAAACTATGG 3’	Rev primer 5’ CTAGTACTGCTGCGACTTGG 3’
Circ_Kif16b	Fwd primer 5’ CAGCTTGTCTATGACCGACC 3’	Rev primer 5’ TGGCGGCTTCCTTCGGATGG 3’
Circ_Ezh2	Fwd primer 5’ GGATACAGCCTGTGCACATC 3’	Rev primer 5’ TCTGAACCTCTTGAGCTGTC 3’
Circ_Ttc3 2-11	Fwd primer 5’ TGCACTTAGCGATGGAAAGAG 3’	Rev primer 5’ TCCTCATAAGGGTAATCTTCCAG 3’
Circ_Sorb2	Fwd primer 5’ CGATCGGAGCCAAGGAGTATT 3’	Rev primer 5’ GATAGTAACTCACTGGACTCACG 3’
Circ_Tcf25	Fwd primer 5’ GAGGACAGCAGTGGGTTCAG 3’	Rev primer 5’ CGAGGATTCTCCTGTGCAGCT 3’
Circ_Bbs9	Fwd primer 5’ GTCTGGAGTAATGCTAATGAGTTG 3’	Rev primer 5’ TGCTTGTGGTTGGTAAACAGCT 3’
Circ_Arhgap32	Fwd primer 5’ GCAAGTTGCAGCGTAATG 3’	Rev primer 5’ TGTCTCATCAGGAACTCCAG 3’
Circ_Txndc1	Fwd primer 5’ GCGATGAAGCCGCTTCTCTAC 3’	Rev primer 5’ GGAAGAGGTCAAGGACCGGA 3’
Circ_Cacna1	Fwd primer 5’ GTGTGGCCTTAGCAATCTATATTC 3’	Rev primer 5’ CCTGGAGTAGGGATGTGCTC 3’
Circ_Strn3 2-4	Fwd primer 5’ GCTATTAAGACAGCACGGATTGC 3’	Rev primer 5’ TCTTCAAATTCTCTTGACCTTTCC 3’
Mmu_Qki 5	Fwd primer 5’ TGTGTTAGGTGCGGTGGCTA 3’	Rev primer 5’ CAGGCAAGCAAAGGCGATTA 3’
Mmu_Qki 6	Fwd primer 5’ ACCCAGTGGTGTGTTAGGTA 3’	Rev primer 5’ AAGGTTTTTCCCGGAGGCTG 3’

Mmu_Qki 7	Fwd primer 5’ GGGCCTGAAGCTGGGTTAAT 3’	Rev primer 5’ CATCCAGCAAGTCAATGGGC 3’
Hsa_Qki	Fwd primer 5’ GCAAAATAGAGGCAAGCCCA 3’	Rev primer 5’ GCGTCTCTGTAGGTGCCATT 3’
Mmu_Hprt	Fwd primer 5’ GCGTCGTGATTAGCGATGAT 3’	Rev primer 5’ TCCTTCATGACATCTCGAGCA 3’

Online Table IV. List of primers used for real-time PCR

Pat.Nr.	Disease	OP	Gender	age	EF	LVEDD
1	ICM	LVAD-IP	m	59	15	60
2	ICM	LVAD-IP	m	45	20	62
3	ICM	LVAD-IP	m	48	10	71
4	ICM	LVAD-IP	m	62	17	65
5	ICM	LVAD-IP	m	62	14	81
6	ICM	LVAD-IP	m	45	20	69
7	ICM	LVAD-IP	m	73	20	69
8	ICM	LVAD-IP	m	56	10	n.v.
9	ICM	LVAD-IP	m	35	n.v.	65
10	ICM	LVAD-IP	m	47	26	52
11	DCM	LVAD-IP	m	44	10	85
12	DCM	LVAD-IP	m	46	19	66
13	DCM	LVAD-IP	m	58	20	78
14	DCM	BVAD-IP	m	38	10	n.v.
15	DCM	LVAD-IP	m	50	15	n.v.
16	DCM	LVAD-IP	m	62	10	90
17	DCM	LVAD-IP	m	62	24	92
18	DCM	LVAD-IP	m	40	10	62
19	DCM	LVAD-IP	m	73	35	42
20	DCM	LVAD-IP	w	58	15	62
1	NF	Spender	w	54		
2	NF	Spender	m	54		
3	NF	Spender	m	43		
4	NF	Spender	m	61		
5	NF	Spender	m	50		
6	NF	Spender	w	55		
7	NF	Spender	m	28		
8	NF	Spender	w	55		
9	NF	Spender	m	52		
10	NF	Spender	m	40		

Online Table V. Human patient details.
